# In Our Mind’s Eye: Thinkable and Unthinkable, and Classical and Quantum in Fundamental Physics, with Schrödinger’s Cat Experiment

**DOI:** 10.3390/e26050418

**Published:** 2024-05-13

**Authors:** Arkady Plotnitsky

**Affiliations:** Literature, Theory, Cultural Studies Program, and Philosophy and Literature Program, Purdue University, West Lafayette, IN 47907, USA; plotnits@purdue.edu

**Keywords:** the cat experiment, classical objects, quantum objects, quantum phenomena, reality without realism (RWR), invisible to thought, visible to thought

## Abstract

This article reconsiders E. Schrödinger’s cat paradox experiment from a new perspective, grounded in the interpretation of quantum mechanics that belongs to the class of interpretations designated as “reality without realism” (RWR) interpretations. These interpretations assume that the reality ultimately responsible for quantum phenomena is beyond conception, an assumption designated as the Heisenberg postulate. Accordingly, in these interpretations, quantum physics is understood in terms of the relationships between what is thinkable and what is unthinkable, with, physical, classical, and quantum, corresponding to thinkable and unthinkable, respectively. The role of classical physics becomes unavoidable in quantum physics, the circumstance designated as the Bohr postulate, which restores to classical physics its position as part of fundamental physics, a position commonly reserved for quantum physics and relativity. This view of quantum physics and relativity is maintained by this article as well but is argued to be sufficient for understanding fundamental physics. Establishing this role of classical physics is a distinctive contribution of the article, which allows it to reconsider Schrödinger’s cat experiment, but has a broader significance for understanding fundamental physics. RWR interpretations have not been previously applied to the cat experiment, including by N. Bohr, whose interpretation, in its ultimate form (he changed it a few times), was an RWR interpretation. The interpretation adopted in this article follows Bohr’s interpretation, based on the Heisenberg and Bohr postulates, but it adds the Dirac postulate, stating that the concept of a quantum object only applies at the time of observation and not independently.

Hamlet:

My father—methinks I see my father.

Horatio:

Where, my lord?

Hamlet:

In my mind’s eye, Horatio.

--William Shakespeare, *The Tragedy of Hamlet*, *Prince of Denmark,* Act 1, Scene 2, ll. 183–185

## 1. Introduction

This article reconsiders E. Schrödinger’s thought experiment, the cat paradox experiment (hereafter “the cat experiment”), and its place in quantum foundations from a new perspective, which removes any paradox from it [1]. So, admittedly, do some other views of the experiment, and Schrödinger himself did not see it as a paradox [1] (p. 157). The present view of the cat experiment is, however, new by virtue of being grounded in a type of interpretation of quantum phenomena and quantum mechanics (QM) that were not previously applied to the cat experiment. This interpretation belongs to the class of interpretations designated here as “reality without realism” (RWR) interpretations, introduced by this author previously [2,3,4,5,6]. The present article, however, focuses more sharply on the role of classical physics (CP) in quantum physics (QP), which is also important to the cat experiment, only briefly commented on in one of these earlier works [2] (pp. 71, 196). The article argues for the necessity of QP in fundamental physics while emphasizing that quantum, as well as relativistic, phenomena, exceed the capacity of classical physical theories (CTs) to explain or predict them. To do so requires quantum theories (QTs) and relativistic theories, respectively. (This article is only concerned with QM and, more marginally, quantum field theory (QFT) in high-energy regimes, in their currently standard forms, and puts aside alternative quantum theories, such as Bohmian mechanics, only mentioned in passing).

RWR interpretations place the ultimate reality responsible for quantum phenomena beyond representation or even conception, thus making this reality literally unthinkable or “invisible to thought”, the expression chosen for the reasons explained below. Quantum phenomena themselves are, by contrast, available to our thinking, “visible to thought”, even to our immediate perception. Accordingly, RWR interpretations define QP, both theoretical, specifically QM or QFT, and experimental, in terms of the relationships between what is thinkable and what is unthinkable; in the case of thinkable, this is in accord with such standard denominations as realist or ontological (or ontic). The present interpretation also assumes that quantum phenomena and the observable part of the instruments used to establish them are described by CP, with the addition of special relativity (SR) in high-energy quantum regimes, handled by QFT. (This assumption is not generally required for an RWR interpretation). As a result, CP becomes an essential part of QP and all fundamental physics—physics that deals with the ultimate constitution of nature. CP also describes the observable parts of the instruments used in relativity, special (SR) or general (GR), or of course in CP itself. In relativity, however, or in CP, all strata of the reality considered are thinkable and, in fact, representable. By contrast, in QP, any observation and measurement of the data obtained in this observation are effects of the interaction between the instruments used and the ultimate reality responsible for what is observed; this reality is unthinkable in RWR interpretations. While co-extensive in CP or relativity, an observation and a measurement are two different procedures in QP, at least in RWR interpretations. In these interpretations, an observation creates a quantum phenomenon through the interaction between the quantum object considered and the instrument used, and then a measurement classically measures the data registered in this observation.

Quantum phenomena are assumed here to be defined by the fact that in considering them (technically, the data found in them), the Planck constant, *h*, which is a classically measurable quantity, must be taken into account. I will put aside the qualifications of this definition (e.g., [2] (pp. 37–38) [7]). These qualifications are not germane to this article, because all quantum phenomena and measurements considered involve *h*, which is essential to the ultimate constitution of nature, assuming that QM and QFT are correct. This is because *h* reflects the Planck scale (10^19^ GeV), the ultimate scale of this constitution, in the present-day understanding of fundamental physics. In the present view, *h*, or any fundamental physical constant, such as *c*, belongs to this understanding, rather than being a property of nature itself. On the other hand, the fact that it is experimentally based also tells us that *h* is the effect of our interaction with nature by means of experimental technology and, thus, depends on something in nature. All equations of QM or QFT used for predicting the data observed in quantum phenomena must contain *h* (or some equivalent constant).

The view adopted in this article restores to CP its position as part of fundamental physics, a position commonly reserved for relativity and QP. While the fundamental nature of the latter theories is maintained by this article, it argues that CP is equally necessary for defining fundamental physics, without of course assuming for CP the same role it had before relativity and QP. It may be true that CP does not deal with the ultimate constitution of nature as both relativity and QP do. Even if this is the case (which may be debated), neither relativity nor QP can, at least in the present view, deal with phenomena considered by them without CP. The latter is necessary for handling both the observed parts of the instruments used and the data obtained by using these instruments. Saying that the role of *h* (defining QP) or, similarly, *c* (as defining relativity), can be disregarded in CP, is different from saying, as is common, that *c* is assumed to be infinite in CP and *h* is equal to zero in CP and relativity. In particular, classical mechanics or electromagnetism need not be, and in the present view, is not, merely assumed to be the limit of QM by making *h* equal to zero or to express in this way Bohr’s correspondence principle. This is correct and allows one to do so in practice, but it is not sufficient for understanding how these theories both differ and relate to each other. Thus, as P. Dirac observed in his first paper on QM, giving a mathematical expression to Bohr’s correspondence principle (explained later in this article): “The correspondence between the quantum and classical theories lies not so much in the limiting agreement when *h* = 0 as in the fact that the mathematical operations on the two theories obey in many cases the same [formal] laws” [8] (p. 315). This view, or the present argument, in no way diminishes the role of *h* in QP or *c* in relativity. This point, instead, is the significance of CP and CT in fundamental physics, including their necessity in both QT and relativity for handling the observations and measurements there. In the present view, CT and QT are two different theories, dealing with two different types of objects, even though classical objects are ultimately composed of quantum objects or (given that, in the present interpretation, the concept of a quantum object is only applicable at the time of observation) the same ultimate reality. It has been argued that, because of this ultimately quantum constitution of classical objects, CT should be seen as merely a (limited) form of QT. In the present view, however, while this view can apply in certain situations, CP is a separate part of fundamental physics, as necessary for dealing with observations and measurements in both relativity and QP. Classical mechanics is not only a special (limited) form of QM, and classical objects are not special types of quantum objects, although quantum objects can sometimes be treated by CT and other times by QT, with important qualifications discussed below.

It follows that, at least as things stand now (an important qualification assumed throughout this article), all three types of theories, classical, relativistic, and quantum, may be (and in the present view are) necessary in fundamental physics, including in QT, which, in the present view, contains both CT and, in high-energy quantum regimes, SR [4]. GR is a separate part of fundamental physics. While GR may play a role in dealing with some quantum phenomena, the *constitution* of quantum phenomena, considered thus far by QT, does not involve gravity. We do not have a QT of gravity, and QFT, which deals with other fundamental forces of nature (electromagnetic, weak, and strong) within the so-called standard model, is incompatible with GR. This incompatibility is one of the main problems of fundamental physics now.

Establishing this role of CP, which is rarely, if ever, done, in mathematical terms, is one of the main contributions of this article. In contrast to the ultimate reality responsible for the data predicted by the mathematics of QT, the reality invisible to thought, in RWR interpretations, this mathematics itself is thinkable and visible to thought, just as is the mathematics of CT or relativity. From its emergence with CT to relativity and QT (the term “classical physics” was introduced in the wake of relativity and QT), modern physics has not only defined itself as a mathematical–experimental science but also made mathematics govern this conjunction. “Modern science is experimental because of its mathematical project”, M. Heidegger asserted in commenting on the origin of modern physics in Descartes and Galileo [9] (p. 93). QM and then QFT required a special type of mathematics, which had not been used in physics previously, such as Hilbert spaces of finite and infinite dimensions, over C, noncommutative (operator) variables, and so forth. As I argue, however, this mathematics cannot be used to predict anything without using CP, describing observed quantum phenomena and the data (over R) they contain.

This argument also allows this article to reconsider Schrödinger’s cat experiment, and reciprocally, use it to support this argument. RWR interpretations have not been previously applied to the cat experiment, including by Bohr, whose interpretation, especially in its ultimate version (he changed his views a few times), was an RWR interpretation. Bohr also argued for the fundamental role of CP in considering quantum phenomena, a view adopted here as the Bohr postulate. It is worth keeping in mind that, while an interpretation of QM commonly involves an interpretation of quantum phenomena, the latter have separate interpretable aspects (noted whenever necessary in this article) which are independent of any theory predicting them.

Eventually, Bohr came to see quantum phenomena as revealing “a novel feature of atomicity in the laws of nature”, “disclosed” by “Planck’s discovery of the quantum of action [*h*], supplementing in such unexpected manner the old [Democritean] doctrine of the limited divisibility of matter” [10] (p. 94). Atomicity and, thus, discreteness or discontinuity in QP initially emerged on this Democritean model, beginning with M. Planck’s discovery of the quantum nature of radiation in 1900, which led him to his concept of the quantum of action, *h*, and A. Einstein’s concept of a photon, as a particle of light, in 1906. The situation, however, especially following the discovery of QM in 1925, revealed itself to be more complex. This complexity led Bohr to the concepts of phenomenon and atomicity, which are essentially equivalent but highlight features of quantum phenomena, such as their individual nature and their discreteness relative to each other. His ultimate RWR interpretation of quantum phenomena and QM was based on these concepts and, correlatively, the role of CP in describing quantum phenomena and the observable parts of the instruments used. (These instruments were also assumed to contain quantum strata, through which they would interact with quantum objects). This interpretation was developed in the late 1930s, following a decade of the development of (and some significant changes in) his views (with a few minor refinements added later). This requires one to specify to which version of his interpretation one refers, which I shall do as necessary, while focusing on his ultimate interpretation, unavoidably in the present interpretation of his interpretation. Unless qualified, “Bohr’s interpretation” will refer to his ultimate interpretation. The designation “the Copenhagen interpretation” requires even more qualifications, beginning with whose interpretation it is, say, that of W. Heisenberg, P. Dirac, J. von Neumann, or that assumed in a given textbook. For this reason, I shall avoid this designation altogether.

The interpretation adopted in this article follows Bohr’s interpretation, as based on two postulates: the Heisenberg postulate, reflecting the role of the unthinkable in QM and, thus, defining RWR interpretations, and the Bohr postulate, reflecting the role of CP in QM, as all observations and measurements in QP are represented by CP. RWR interpretations based on the Heisenberg postulate alone, without assuming the Bohr postulate, are possible. Conversely, the Bohr postulate need not be limited to RWR interpretations and can be assumed by realist interpretations. For the reasons explained below, the present interpretation adds a third postulate, the Dirac postulate, according to which the concept of a quantum object is only applicable at the time of observation. For clarity, I will restate all three postulates, which guide the arguments of this article:

(1) The Heisenberg postulate, most essentially defining “reality without realism” (RWR) interpretations, states that the reality ultimately responsible for quantum phenomena is beyond representation or even conception.
(2) The Bohr postulate states that observations and measurements used in QP and defining quantum phenomena are represented by CP, thus giving CP the fundamental role in QP.
(3) The Dirac postulate states the concept of a quantum object is only applicable at the time of observation, and not independently.

All three postulates are assumptions that could be falsified, although a falsification of an interpretation is not the same as (and is more complex than) a falsification of a theory by experimental evidence. The latter is not at stake in this article, which assumes both QM and QFT to be correct, as things stand now.

The Heisenberg postulate was, in effect, introduced by Bohr’s 1913 atomic theory, in considering the transitions, “quantum jumps”, as they were called then, between stationary states of electrons (accompanied by an emission or absorption of quanta of radiation, *h*ν). Bohr’s theory still retained a realist view of stationary states by assuming them to be represented as orbits of electrons around nuclei. The term “jump” is misleading in suggesting some representation of what happens. Electrons do not jump; their states discontinuously change, and no representation of how they do this is available, at least in RWR interpretations. Bohr’s concept of “quantum jump” was, thus, accompanied by a subtle but crucial shift from representing, even probabilistically, the motion of electrons, as in the preceding, such as Lorentz’s, electron theory, by the discrete transitions between states of electrons [2] (pp. 88–90). (I am indebted to L. Friedel on this point, made, in an unpublished paper, in conjunction with QM, rather than Bohr’s 1913 theory, where, I think, it originates). This shift became central to Heisenberg’s discovery of QM in 1925, thus defining RWR interpretations of quantum phenomena and QM by the discrete transitions between the states of electrons, without assuming any classical-like motion of electrons at all. In Heisenberg’s approach, electrons did not move in orbits around nuclei either: their quantum states (associated with variables other than energy, which remained the same in a stationary state) discontinuously changed. These changes, moreover, were only observed, classically, as discrete phenomena, in observational instruments, and probabilistically predicted by QM. So, Heisenberg, in effect, assumed the Bohr postulate.

In both Bohr’s 1913 theory and Heisenberg’s QM, the emergence of quantum phenomena was only assumed to be beyond representation or knowledge, in which case, I shall speak of a *weak* RWR interpretation, rather than beyond conception, in which case, I shall speak of a *strong* RWR interpretation. I am primarily concerned here with strong RWR interpretations, moreover, only those (there can be others) that assume the Bohr postulate. (Unless qualified, RWR interpretations will refer to these RWR interpretations. By contrast, the use of the Dirac postulate, which is not commonly assumed, will be noted throughout). The first strong RWR interpretation was offered by Bohr as the ultimate version of his interpretation, around 1937, following the Einstein–Podolsky–Rosen (EPR) paper [11], to which Bohr replied [12]. Schrödinger’s cat paradox paper was also a reply to the EPR paper. (All three papers were published in 1935). While Bohr was aware of Schrödinger’s paper, it is not clear how well he knew it. He does not appear to have ever commented on it or the cat experiment. On the other hand, as discussed in Section 3, Schrödinger had read the draft of Bohr’s reply, while working on his cat paradox paper.

In contrast to the RWR view, the realist view is defined by the assumption of the possibility of either representation or more limited knowledge or at least a conception of how the phenomena considered are possible. While the conception is much older in philosophy, with its history reaching as far back as Plato and Aristotle, in modern physics, as a mathematical–experimental science, it originates with classical mechanics. As emphasized by Bohr and Heisenberg, the concepts of classical mechanics emerged as mathematized refinements of our daily concepts—concepts arising from our phenomenal experience of the world. In Aristotle’s physics, physical concepts were *philosophical* refinements of this experience, although it is possible to argue that there were elements of mathematization even there [13,14,15]. All modern physics is defined by suitably mathematized idealizations of physical reality. This connection between physical and daily concepts has proven to be difficult to use in QT, even in realist interpretations, which assume that QM or QFT provides a mathematized representation of quantum objects and processes. Such a representation is no longer a mathematical refinement of our general phenomenal experience found in CP, although QM or QFT formally adopts some mathematics used in CP.

Several additional clarifications are in order. By CP, I mean (as Bohr appears to have done, although he rarely specified it) such theories as classical mechanics, classical statistical physics, classical electromagnetic theory, or chaos theory, not available during Bohr’s lifetime, and the phenomena these theories consider. By classical mechanics, I refer to Newton’s mechanics defined by its three main laws, the law of inertia, the law of the changes a force can have on the motion of a body, *F = ma*, and the law of action and reaction between interacting bodies, as equal in magnitude but opposite in direction. These theories and the phenomena they consider are sufficient for the present purposes of representing the observable part of the instruments used in QP and the view of CTs and CP adopted here.

It is, in principle, possible to give a (more) rigorous formal definition, in terms of a single feature or a determinate set of features, of the difference between CTs and QTs. There have been several approaches to doing so, although there are debates concerning most of them, which is not surprising given the subtlety of this task. The situation is, as noted, equally complex in defining the difference between classical and quantum phenomena, which is not the same as defining their theories, as there can be more than one in each domain. Approaches arising from Schrödinger’s concept of entanglement—which Schrödinger himself sees as a uniquely characteristic trait of QT vs. CT, or “classical lines of thought” in developing a physical theory—are especially compelling to this author [1] (p. 157). Entanglement is not a feature of quantum phenomena but of QM, as a theory predicting quantum phenomena, including, by using entanglement, those containing quantum correlations. The concept also reflects the mathematics of QM (or QFT) as fundamentally different (including as defined over C) from that of CT or relativity. Schrödinger’s explanation of entanglement in the cat paradox paper is too elaborate to cite. I shall instead cite his companion paper:

When two systems, of which we know the states by their respective representatives, enter into temporary physical interaction due to known forces between them, and when after a time of mutual influence the systems separate again, then they can no longer be described in the same way as before, viz. by endowing each of them with a representative of its own. I would not call that *one* but rather *the* characteristic trait of quantum mechanics, the one that enforces its entire departure from classical lines of thought. By the interaction of the two representatives have become entangled. Another way of expressing the peculiar situation is: the best possible knowledge of a *whole* does not necessarily include the best possible knowledge of all its *parts*, even though they may be entirely separate and therefore virtually capable of being ‘best possibly known,’ i.e., of possessing, each of them, a representative of its own. The lack of knowledge is by no means due to the interaction being insufficiently known—at least not in the way that it could possibly be known more completely—it is due to the interaction itself. [16] (p. 555)

Schrödinger’s point that “the lack of knowledge is by no means due to the interaction being insufficiently known—at least not in the way that it could possibly be known more completely—it is due to the interaction itself” is consistent with QM in RWR interpretations. Schrödinger, who shared Einstein’s discontent with QM, was no champion of the RWR-type view of QM, or of QM itself as allowing for or even being conducive to this view. In strong RWR interpretations, the expression “the lack of knowledge” is not rigorously applicable. At stake in these interpretations is not the lack of knowledge that could, in principle, be obtained or even an assumption of anything conceivable to the thought that is responsible for the situation located by Schrödinger and for quantum phenomena. In fact, as Schrödinger realized, any quantum phenomenon can be considered in terms of an entanglement between the quantum object under investigation and the observational instrument used, thus enabling predictions concerning the outcome of future quantum experiments [2] (pp. 192–196). A compelling formal definition of the difference between CT and QT along these lines (in quantum information theory) is given in [17], by formalizing Schrödinger’s point as the “purification postulate”. (Important implications of this definition for the question of falsification in QT vs. CT are considered in [18]). As “*the* characteristic trait” of entanglement that Schrödinger locates in QM, the purification postulate is strictly mathematical. As such, it presupposes a certain mathematical structure, defined over C, although it does not assume a Hilbert space, as is more common in such projects, following von Neumann [19]. This presupposition itself represents a *characteristic* trait of QT, especially in RWR interpretations: what distinguishes QT from CT is not defined by a physical concept or concepts (as in the case of relativity vs. CP), subsequently represented mathematically, but rather by an abstract mathematical structure.

Quantum phenomena are never entangled because any observed quantum phenomenon arising from and predicted by using an entanglement will disentangle it. Speaking of entangled quantum objects requires caution as well because the combined entangled system (as a whole) precludes sufficient knowledge of each part to define each object [1] (p. 161). In the present view, moreover, by the Dirac postulate, a quantum object is only defined at the time of observation. This makes entanglement a feature of QT, enabling certain specific predictions concerning quantum objects, defined when the corresponding experiment is performed, on the basis of certain specific previous experiments on quantum objects, defined at the time of these previous experiments [2] (pp. 262–263). Nevertheless, the existence of quantum correlations, at stake in Bell’s, the Kochen–Specker, and related theorems, is an established experimental fact. Thus, one is also dealing with nature or, which is a necessary qualification in the present view, our interactions with nature by means of specific technology. There is no QP without quantum instruments because the ultimate reality responsible for quantum phenomena is only manifested in its effects observed in these instruments and never otherwise, specifically not by our sense perception. The latter is sometimes sufficient for observation in CP, but never in QP. Quantum instruments are understood here as technological devices capable of registering such effects. A QT, such as QM or QFT, is able to predict these effects by using our “mathematical instruments”, as Bohr called them (referring to their abstract character as “higher algebra”), in parallel with measuring instruments [20] (v. 1, p. 51). Quantum entanglement is part of the mathematical technology of QM. It is conceivable, however, that quantum correlations could be predicated by a QT that does not contain the mathematics of entanglement.

By the Heisenberg postulate, defining RWR interpretations, how quantum phenomena come about is not represented by QM or QFT, but only predicted by it, in general probabilistically. Thus, in these interpretations, QM or QFT has no physical connection, apart from these predictions, to either the ultimate nature of reality responsible for quantum phenomena or, if one assumes the Bohr postulate, to quantum phenomena, because the latter are described by CP. Accordingly, why the mathematics of QM or QFT can predict the outcomes of quantum experiments, even if only probabilistically (which is, however, strictly in accord with what is observed) is in turn beyond knowledge or even conception. We know how the mathematics works, but we do not know, and perhaps cannot know (or even conceive) of why it works.

By contrast, in CP or relativity (SR and GR), the mathematical formalism represents, as a mathematized idealization, the physical reality responsible for the phenomena considered and connects these phenomena by continuous and classically causal processes. The concept of classical causality is defined in Section 2 (where I also explain the qualification “classical”, which is not standard). Briefly, classical causality is defined by the claim that the state, *X*, of a physical system, is determined, in accordance with the law, at all future moments once its state, *A*, is determined at a given moment, and state *A* is determined by the same law by any of the system’s previous states. The phenomena considered in CP and relativity can, moreover, be identified, for all practical purposes, with the physical objects considered, because the interference of measuring instruments can be neglected [20] (v. 1, p. 53). This identification is no longer possible in considering quantum phenomena, in the constitution of which the role of observational instruments, precluding this identification, is irreducible. Nobody has ever seen a moving electron or photon. It is invisible to observation and, in strong RWR interpretations, is beyond conception, and is invisible to thought. It is only possible to observe traces of their interactions with measuring instruments—traces that are “visible” even to our immediate perception. (Such traces may also be “clicks” that we hear rather than see). These traces make it difficult and, in strong RWR interpretations, impossible to reconstitute the ultimate nature of the reality responsible for them, whether one understands this reality in terms of quantum objects or assumes, by the Dirac postulate, that a quantum object is an idealization applicable only at the time of observation.

Either way, the situation entails an unavoidable discrimination between quantum objects and instruments and, hence, observed phenomena. This discrimination, according to Bohr, “may indeed be said to form a *principal distinction between classical and quantum-mechanical description of physical phenomena*” [12] (p. 701). This statement does not mean that QM describes these phenomena, it only, probabilistically, predicts them, while, as phenomena, they are physically described by CP. By “the quantum-mechanical description”, Bohr refers to the overall structure of QT, which includes CP (in describing observed quantum phenomena), cum its interpretation, in this case, an RWR interpretation. This discrimination also precludes, regardless of interpretation, the identification, even for practical purposes, of quantum phenomena with quantum objects, possibly in classical physics and relativity.

The present interpretation further stratifies this structure of observation in QP by the Dirac postulate, which states that the concept of a quantum object is only applicable at the time of observation, but not to anything assumed to exist independently in nature. Bohr’s argumentation might be seen at certain points, suggesting the Dirac postulate. Bohr, however, never stated this type of postulate. Related suggestions have been made throughout the history of QM and QFT, but not from the RWR perspective. One such suggestion was made by Schrödinger, who thought that a particle should not be seen as something permanent or existing by itself but is “an instantaneous event” [21] (p. 133). Schrödinger, however, advanced this idea under the realist assumption of the continuous field-like underlying ultimate reality, which would be represented by a QT (not necessarily QM). In strong RWR interpretations, the ultimate reality responsible for quantum phenomena is beyond thought and, hence, cannot be assumed to be continuous, any more than discrete. In Bohr’s interpretation in all its versions, the ultimate reality responsible for quantum phenomena appears to have been associated with quantum objects, as independent RWR-type entities, irreducibly different from quantum phenomena. A quantum object is a physical object responsible for the existence of a quantum phenomenon, as an effect of the interaction between this object and the observational instrument used. As will be seen, it is possible in a quantum experiment to consider the object under investigation by means of QP as an object that also contains a classical part, such as the cat in the cat experiment. In this case, however, this composite object must contain a properly quantum object, such as a radioactive atom capable of emitting a quantum particle in the cat experiment, for the observed phenomenon to be a quantum phenomenon. The cat itself cannot be considered a *properly* quantum object; it cannot be treated by QM if not physically connected to a properly quantum object—an object that can be treated by QM (or QFT) *independently* of any other object. The cat cannot. Considered by itself, it can only be treated by CP. The Dirac postulate could only apply to properly quantum objects, and not to any classical object, such as a cat.

My use of the designation “Dirac postulate” originates from Dirac’s discovery of antimatter, one of the greatest discoveries of twentieth-century physics. Unlike the Heisenberg postulate by Heisenberg and the Bohr postulate by Bohr (even without using these designations), this postulate was not considered by Dirac. It may, however, be seen as having been first suggested by Dirac’s equation for the (free) relativistic electron:

$$\left(\beta mc^{2}+\sum_{k=1}^{3}\alpha_{k}p_{k}c\right)\psi \left(x,t\right)=i\hbar \frac{\partial \psi (x,t)}{\partial t}$$

$$\;\;\;\;\;\;\;\;\alpha_{i}^{2}=\beta_{i}^{2}=I_{4}\;I_{4}\;\mathrm{is}\;\mathrm{the}\;\mathrm{identity}\;\mathrm{matrix}$$

$$\alpha_{i}\beta +\beta \alpha_{i}=0\;\alpha_{i}\alpha_{j}+\alpha_{j}\alpha_{i}=0$$

Here, $\psi \;\left(x,\;t\right)$ is the wave function of the electron with rest, *m*, and space and time coordinates, *x*, *t*. The wave function is explained below. Briefly, it assigns a complex quantity to each point, *x,* and each time, *t*. In Born’s interpretation, it is associated with the probability amplitude, defining the wave function as providing the probability density by the square modulus of |$\left.\psi \right\rangle$, ${\left\|\psi \right\|}^{2}$. The latter quantity is always a positive real number and can be normalized so that the probability is between zero and one. Moreover, *p*_1_, *p*_2_, and *p*_3_ are the components of the momentum, in the sense of the momentum operator in Schrödinger’s equation (considered below). The new elements in Dirac’s equation are the four 4 × 4 matrices, *α*_1_, *α*_2_, *α*_3,_ and *β*, and the four-component *ψ*, necessary because the evaluation of *ψ* at any given point in the configuration space is a “bispinor”. The name is used because *ψ* is a superposition of Hilbert space elements that allow one to make predictions of possible outcomes of measurements as a spin-up electron, a spin-down electron, a spin-up positron, and a spin-down positron. Dirac’s equation contains spin automatically, in contrast to QM, where predicting the spin needs to be handled separately by using Pauli (2 × 2) matrices. The wave function *ψ (t*, *x)* takes value in a Hilbert space *X* = C^4^ (Dirac’s spinors are elements of *X*). For each *t*, *y (t*, *x)* is an element of *H* = L^2^ (R^3^; *X*) = L^2^ (R^3^) ⊗ *X*.

A key new structural feature of this equation was that, while symmetrical (as required by relativity), concerning space and time, it contained only first-order derivatives in both space and time, vs. its predecessor, the Klein–Gordon equation, which contained the second-order derivatives in both. Schrödinger’s equation, which was not relativistic and did not need to be symmetrical in space and time, contained the second-order derivative in space and the first-order derivative in time. Its first-order derivative in time was the main reason for the same feature of Dirac’s equation. Otherwise, the nonrelativistic limit of Dirac’s equation could not be Schrödinger’s equation. Establishing that this was the case was imperative for Dirac’s theory. (One of the problems of the Klein–Gordon equation was that this was not the case for it). I have considered Dirac’s equation in detail on several previous occasions (e.g., [2] (pp. 159–164)).

Now, while originally designed for an electron, the equation revealed itself to be an equation for both the electron and the positron. Dirac’s equation reflected and, in fact, led to the discovery that the identity of a particle type could no longer be assumed in successive observations, as in CP or even nonrelativistic QT: the initial observation can register an electron, while the next one can register a positron or a photon, or an electron–positron pair, with the probabilities defined by the same equation. Once one moves to still higher energies, the panoply of possible outcomes becomes greater. Depending on how high the energy is, one can find any known elementary particle or a combination of them, that is, the corresponding effects will be registered. The Hilbert spaces and operator algebras involved acquire more complex structures, linked to the appropriate Lie groups and their representations, defining (when these representations are irreducible) different elementary particles. In the case of QED, we only have electrons, positrons, and photons, single or paired; in QFT, depending on how high the energy is, one can literally find any known and possibly as yet unknown elementary particle, or a combination thereof, with the situation further complicated by the role of virtual particles, or something in nature that compels some to introduce the latter concept [2] (pp. 273–306) [3]. Although, like anything quantum, these transformations can only be handled probabilistically, they also have a complex ordering to them. Thus, in addition to correlational patterns akin to those found in low-energy (QM) regimes, they obey symmetry principles, especially local (gauge) symmetries. The latter has been central to QFT, not the least in leading to discoveries of new particles, such as quarks and gluons inside the nucleus, and then various types of them, eventually establishing the standard model of particle physics, with all known fundamental forces of nature, apart from gravity. QED is an abelian gauge theory with the symmetry group U(1) and has one gauge field, with the photon being the gauge boson. The standard model is a nonabelian gauge theory governed by the tensor product of three symmetry groups U(1) ⊗ SU(2) ⊗ SU(3) and broken symmetries. It has twelve gauge bosons, namely, the photon, three weak bosons, and eight gluons. SU(2) is the symmetry group of the QFT of the weak force (unified with QED), and SU(3) is that of quantum chromodynamics (QCD), a QFT of the strong force, which is, thus far, a separate theory. This situation invites adopting the Dirac postulate. There are, however, reasons to use it in low-energy quantum regimes, including in view of paradigmatic quantum experiments, such as the double-slit experiment [5]. Some of these reasons will become apparent as this article proceeds.

The next section outlines the RWR interpretation adopted in this article, cast in terms of the relationships between what is visible and invisible to thought. Section 3 discusses the letter exchange between Schrödinger and Bohr concerning the role of classical concepts in QT. Section 4 considers Schrödinger’s cat experiment from the perspective established in Section 2 and Section 3.

## 2. Reality without Realism: Visible and Invisible to Thought in Fundamental Physics

RWR interpretations imply that modern physics, as a mathematical–experimental science, with mathematics defining this conjunction, contains two types of theories or interpretations of theories. These types will be designated as, respectively, “realist” or “ontological”, and RWR, the only type of theories or interpretations that are not realist that will be considered here. The terms “realist” and “ontological” are commonly close in their meanings and will be used, as adjectives, interchangeably here. I shall adopt “realism”, as a noun, as a more general term, and use “ontology” to denote the representation or conception of the reality considered by a given theory. (Another common term for a realist theory is “ontic”, coming—as with ontological—from the ancient Greek *on* [Being]). It is more rigorous to see a different interpretation of a given theory as forming a different theory, because an interpretation may involve concepts not shared by other interpretations. For simplicity, however, I shall continue to speak of different interpretations of QM or QFT. On the other hand, in the case of CT, comprised of several theories, and relativity (SR or GR, which contains different versions), I shall refer to the theories themselves, because most interpretations of these theories, including those assumed here, are realists. Some qualifications concerning these interpretations are required and will be made later. Also, as QP or CP, relativity is not restricted to theories, SR and GR, and I shall qualify when I refer to relativistic phenomena rather than to SR or GR.

I provide the basic definitions of realist and RWR theories or interpretations. Realist theories or interpretations aim to represent, or at least offer a conception of, the physical reality considered at all levels, primarily by mathematized models, idealizing this reality. Such a representation or (in strong RWR interpretations) even conception is not possible in RWR interpretations of QM or QFT in the case of *the ultimate reality responsible for quantum phenomena*. This impossibility defines RWR interpretations. I qualify because RWR representations allow for and entail realist representations at other levels, including in considering quantum phenomena, represented (by the Bohr postulate) by CP.

As are most concepts of reality in realist theories, the concept of RWR is based on more general concepts of reality and existence, which are assumed here to be primitive concepts and are not given analytical definitions. By “reality” I refer to that which is assumed to exist, without making any claims concerning the *character* of this existence or reality, claims that, as explained below, define realism. The absence of such claims allows one to place this character beyond representation or even conception, as in RWR interpretations. I understand existence as a capacity to have effects on the world. The assumption that something is real, including the RWR type, is made, by inference, based on such effects, as it must be to be rigorous. RWR interpretations are based on the idea that observable effects of physical reality allow for a representation of these effects but not necessarily a representation or even a conception of how they are possible. Such a representation or conception is not possible in RWR interpretations in the case of the ultimate reality responsible for quantum phenomena, making this reality invisible to thought in strong RWR interpretations. The assumption of the independent existence of nature or matter essentially amounts to the assumption that it existed before we existed and will continue to exist when we no longer exist. This assumption has been challenged to the point of denying that there is any material reality. Plato and Bishop Berkeley are the most famous cases, respectively, ancient and modern, of this denial. It is true that any conception of how anything exists, or even that it exists, including as beyond thought, still belongs to thought, as do all our concepts and theories. It does not follow, however, that something beyond thought does not exist. That we cannot imagine an entity that is neither continuous nor discrete does not mean that such an entity does not exist in nature. This point was made over a century ago when considering similar complexities in set theory by H. Lebesgue, one of the founders of the modern measure and integration theory [22] (pp. 261–273). The concept of RWR allows for such a possibility.

Extending the concept of RWR beyond QP, which is possible [2] (pp. vii–xxiv), implies that all our theories, including CT, relativity, and QT, are creations of our thought in our interaction with the world. This interaction is expressly manifested technologically in QP or relativity, as either theory requires a specific experimental technology to observe, in QP technologically to *construct*, the phenomena considered. But this interaction is also found in CP because all classical phenomena considered, as well as CT, are still created by thought [2] (pp. vii–xxiv). Nature itself, as existing independently of us, is in the present view, a form of RWR, and as such is no more classical than relativistic or quantum. Fortunately for us, however, our *interactions* with nature in physics can be handled by CP, relativity, or QP, or by natural sciences in other domains. It is uncertain how well we will fare in extending fundamental physics, as we need to do, for example, in bringing some harmony between gravity, handled by GR, and other fundamental forces of nature, handled by QFT (within the standard model). It is also uncertain what kind of theory such—a theory, if ever found, will be, for example, whether it will be quantum in the current sense.

(A) There is still the question of whether our inability to conceive of the ultimate nature of reality responsible for quantum phenomena is related to one or the other of the following two possibilities. This inability characterizes the situation in QP as things stand now, while allowing that quantum phenomena or whatever may replace them will, at some future point, no longer make this assumption necessary and, thus, no longer make RWR-type interpretations viable, reverting to a realist view.
(B) This inability reflects the possibility that this reality will never become available to thought.

Logically, once (A) is the case, (B) is possible but is not certain. There does not appear to be any experimental data compelling one to prefer either (A) or (B). (A) and (B) are, however, different in defining how far our mind can, in principle, reach in understanding nature. The strong RWR view is not about replacing what is unthinkable with new creations of thinking. Doing so, or replacing what is unknown with new knowledge, is of course an important aspect of mathematical and scientific thought, including when the RWR view is adopted. The strong RWR view is, however, about creating new forms of thought, while assuming that there is something, a reality, in nature or in mind (as in mathematics), which is beyond the reach of thought and will never be reached by thought as suggested by Lebesgue. There might be things in nature or thought (it follows our unconscious thought), including mathematical things, that our thought, conscious or unconscious, cannot reach. This is not merely a matter of philosophy, because physics or, as Lebesgue thought, mathematics may be affected by this epistemology, as this “beyond-thought” is responsible for entities, physical and mathematical, which thought, such a theory, must deal with [22] (pp. 261–273). This is the main reason to distinguish these views and for this author to see (B) as possible, although much of my argument here applies to both, but not all of my argument. Bohr at least assumed (A), and some of his statements—especially those that are stronger than interpretive claims concerning our lack of access to the ultimate nature of reality responsible for quantum phenomena—suggest that he might have entertained (B).

The qualification “as things stand now” applies, however, to (B) as well, even though it might appear otherwise given that this view precludes any conception of the ultimate constitution of the reality responsible for quantum phenomena not only now but also in the future. It still applies because a return to realism in QT is possible, if QT, as currently constituted, is given a more definitive realist interpretation, or is replaced by an alternative theory that requires a realist interpretation. Either eventuality might make assuming (B) as obsolete, even for those who hold it in favor of a realist view, without, however, abolishing the concept of RWR as such, which may be applicable elsewhere.

These considerations also provide a better perspective on the ultimate nature of the Bohr–Einstein debate, or (they are parallel) the Bohr–Schrödinger debate, discussed below. Neither debate was primarily about what QM could or could not do, on which their disagreement, while not discountable, was not so crucial. Both debates were ultimately about whether nature could allow for a “better” or “more complete” theory of quantum phenomena, according to Einstein’s or Schrödinger’s, essentially realist, criteria for such a theory. They thought, in accord with (A) above, that nature *should* allow for such a theory, and it is possible (I am not certain) that Bohr thought that it *will not*, rather than *may not*. In any event, the present position is that it *may not*, which is not the same as it *will not*. Admittedly, the RWR view is human, just as was that of Einstein or Schrödinger. The difference is that, contrary to Einstein’s, Schrödinger’s, or others’ positions advancing realism as imperative in fundamental physics, the RWR view is not seen here as imperative. It is only assumed here to be practically justified, as things stand now, and possibly to remain to be practically justified in the future. As stated above, however, it is also possible that this view, even in its stronger (B) version, will become obsolete one day.

Theories or interpretations of each type, realist and RWR, treat the reality considered differently concerning the *ultimate nature* of this reality, assumed to be representable and, thus, *visible to thought* in realist theories, to be beyond representation or even conception and, thus, *invisible to thought* in RWR ones. At the same time, RWR interpretations assume that CP (cum SR in high-energy regimes) and, hence, realism, apply at some levels of the reality considered, specifically, by the Bohr postulate, to the observable parts of the instruments used and, thus, to the representation of quantum phenomena by CTs. The latter, however, cannot predict what is observed in these phenomena. This is an automatic consequence of the uncertainty relations. Suppose one measures the position of the quantum object at time *t*_1_ and then wants to use classical mechanics to predict its position at a future time, *t*_2_.CT, however, can do so, and can do so ideally exactly, only if one knows the momentum of the object at *t*_1_, which is impossible by the uncertainty relations. Is there a theory that can still make this prediction? Yes, luckily for us, QM, can, while respecting the uncertainty relations. It can, however, only do so by predicting the probability of finding the object in a given area. (Note that Bohmian mechanics that can also make such predictions, equally respecting the uncertainty relations, is not a CT, even though it is realist and classically causal, at the cost of its nonlocal nature in the sense of being inconsistent with SR). In fact, relativity (SR or GR) also assumes that the observed parts of the instruments used (rods and clocks) are described by CP in each local frame of reference. All physical reality considered is, however, representable by either relativity or, in the case of locally observed phenomena, CP.

The existence of the RWR-type reality ultimately responsible for quantum phenomena is inferred from the character of the data observed in quantum experiments, such as, paradigmatically, the double-slit experiment. These data, along with the mathematics of QT, are visible to thought and unambiguously communicable, and in this sense, objective, just as are the data of CP and the mathematics of CT or the data and mathematics of relativity. These facts, along with the predictive capacity of QT, enable QP to function as a mathematical–experimental science, extending the project of modern physics.

Most realist theories are representational. They aim at representing the reality they consider, in modern physics by mathematized models, suitably idealizing this reality. It is possible to aim, including in QT, for a strictly mathematical representation of this reality apart from physical concepts, at least as they are ordinarily understood, say, in CP or relativity. It is also possible to assume an independent structure (defined by properties and relationships among them) of the reality considered, while admitting that it is either (A) not possible to represent this structure or (B) even to form a rigorously specified concept of it, either at a given moment in history or even ever. Under (A), a theory that is merely predictive could be accepted for lack of a realist alternative, usually with the hope that a future theory will do better by being a representational theory. Einstein held this view of QM. What, then, grounds realism most fundamentally is the assumption that the ultimate constitution of reality possesses properties and the relationships between them, or, as in (ontic) structural realism, just a structure, the elemental constituents of which are not defined in terms of properties [23]. Such properties or relationships may either be ideally represented and, hence, known, or be unrepresentable or unknown or even unknowable. They are, however, still assumed to be conceivable, usually with a hope that they will eventually be so represented. As pertaining to physics, the concept of realism just outlined is sometimes called “scientific realism”.

Thus, classical mechanics (used in dealing with individual objects and small systems, apart from chaotic ones), classical statistical mechanics (used in dealing, statistically, with large classical systems), chaos theory (used in dealing with classical systems that exhibit a highly nonlinear behavior), or relativity (SR and GR) are realist theories. Classical statistical mechanics does not represent the overall behavior of the systems considered because their mechanical complexity prevents such a representation. But it assumes that the individual constituents of these systems are represented by classical mechanics. In chaos theory, which, too, deals with systems consisting of large numbers of atoms, one assumes a mathematical representation of the behavior of these systems. Relativity posed insurmountable difficulties for our general phenomenal intuition because the relativistic law of addition of velocities (defined by the Lorentz transformation) in SR, $s=\frac{v+u}{1+(vu/{c)}^{2}}$, for collinear motion, runs contrary to any possible intuitive conception. We cannot conceive of this kind of motion, thus, making this concept of motion no longer a mathematical refinement of a daily concept of motion as the concept of motion is in CP. Relativity was the first physical theory that defeated our ability to form a visualization of an elementary individual physical process, although the concept of (classical) field in Maxwell’s electromagnetism already posed complexities in this regard. Bohr did not miss this point in noting “the great significance of Einstein’s theory of relativity in recent development of physics with respect to our emancipation from the demands of visualization” [20] (v. 1, pp. 115–116). “Emancipation” is not a casual word choice, rarely found in Bohr’s writings, known for their deliberate choices of words. SR or GR, however, still offers a mathematically idealized representation of the reality considered, thus making this reality visible to thought, even if not our daily phenomenal intuition.

QP brought this emancipation to the level of the invisible to thought, as mathematically reflected in the Hilbert space or other versions of formalism as defined over C. (All these versions, thus far, are defined over C or are equivalent to being so defined). The mathematics itself poses difficulties in seeing this formalism as representing anything physical in space and time, always represented by concepts or quantities over R. The formalism of QM or QFT relates (via Born’s rule) to all observed quantities considered by the means of probabilistic predictions of the outcome of the measurement. As Bohr noted in 1927, “the symbolic character of Schrödinger’s method appears not only from the circumstance that its simplicity, similarly to that of the matrix theory [of Heisenberg], depends essentially upon the use of imaginary arithmetic quantities” [20] (v.1, p. 76). QM and then QFT are mathematically continuous theories (over C) able, via Born’s rule, to predict probabilities of the real numerical outcomes of always discrete events, fully in accord with the experimental evidence, still in place now. The ultimate reality responsible for quantum phenomena is, in strong RWR interpretations, beyond the reach of thought and, thus, cannot be assumed to be either continuous or discrete.

As Bohr stated in the same article, known as the Como lecture, which presented his first interpretation of QM, in CP and relativity “our … description of physical phenomena [is] based on the *idea* that the phenomena concerned may be observed *without disturbing them appreciably*” [20] (v. 1, p. 53; emphasis added). This lack of disturbance also enables one to identify these phenomena with the objects considered. By contrast, “any observation of atomic phenomena will involve an *interaction [of the object under investigation] with the agency of observation* not to be neglected” [20] (v. 1, p. 54; emphasis added). One should note the subtle nature of this contrast: the interaction between the object under investigation and the agency of observation *gives rise* to a quantum phenomenon rather than *disturbs* it [20] (v. 2, p. 64). Relativity represented a step in this direction, as, in contrast to Newtonian mechanics, space and time were no longer seen as preexisting (absolute) entities then measured by rods and clocks. They were instead defined by rods and clocks in each reference frame. Still, the interference of observational instruments in the behavior of the objects considered could be disregarded, thus, for all practical purposes, allowing the identification of these objects with observed phenomena and considering them independently of observation. Disregarding this interference is no longer possible in considering quantum phenomena, empirically, as things stand now, regardless of interpretation, and, hence, in realist interpretations of QM, or in alternative theories, such as Bohmian mechanics. As indicated above, it may not be rigorously possible even in CP or relativity because all phenomena considered, as well as our theories, are still created by our thought [2] (pp. vii–xxiv). Bohr is careful to refer to “the idea” and, hence, the assumption “that the phenomena concerned may be observed without disturbing them appreciably”, rather than saying that such is, in fact, the case. This assumption is, however, workable in CP and relativity for all practical purposes.

Kant might have had in mind something similar in allowing Euclidean geometry and Newtonian mechanics to be objectively true representations of the ultimate constitution of nature [24] (p.115) [2] (pp. xiv–xv, 57–58). Otherwise, it would be difficult to reconcile the difference between things-in-themselves in nature and phenomena as appearances, including those representing nature, in our mind. The definitive interpretation of Kant on this point is difficult. Bohr, on the other hand, is clear. In QP or relativity, one always needs experimental technology beyond human bodies to handle the phenomena considered. In QP, this technology is part of and sometimes *is* “the agency of observation”, in principle, precluding identifying the phenomena observed with quantum objects. This impossibility still allows for realist interpretations of QM or QFT or alternative realist theories. On the other hand, it opens the possibility of RWR interpretations of QM or QFT, or quantum phenomena themselves.

The reasons for my emphasis on visible and invisible, extended to the idea of visible and invisible to thought, are both theoretical and historical. Theoretically, our capacity for visualizing the world, defined by the neurological functioning of our brain (about 60% of which deals with vision), is crucial to our thinking. This capacity has shaped CP as a mathematical refinement of the world we observe, but was defeated, first by SR, and then by QT. The latter brought into physics the possibility of considering that is beyond the reach of thought altogether. Historically, this emphasis follows Bohr’s appeal to the impossibility of visualization of the ultimate reality responsible for quantum phenomena. Bohr’s use of “visualization” was in part due to the German term for intuition, *Anschaulichkeit*, which etymologically relates to what is visualizable, “shown” to thought.

Even before, albeit only by a few months, Heisenberg’s discovery of QM, based on “abandoning the ordinary spacetime description” [20] (v. 1, p. 48) and, hence, on a (weak) RWR interpretation, Bohr said:

I am forcing myself these days with all my strength to familiarize myself with the mysticism of nature and am attempting to prepare myself for all eventualities, indeed even for the assumption of a coupling of quantum processes in separated atoms. However, the costs of this assumption are so great that they cannot be estimated within the ordinary spacetime description. [25] (v. 5, pp. 79–80, p. 237, Letter to Heisenberg, 18 April 1925)

In a letter to M. Born, a few days later, he added:

[Quantum experiments] preclude the possibility of a simple description of the physical occurrences by means of visualizable pictures…. [S]uch pictures are of even more limited applicability than is ordinarily supposed. This is of course almost a purely negative assertion, but I feel that … one must have recourse to symbolic analogies to an even greater extent than hitherto. Just recently I have been racking my brain to dream up such analogies. [25] (v. 5, p. 311, Letter to Born, 1 May 1925)

The word “mysticism” will soon disappear from Bohr’s writings, replaced by an emphasis on QM as a rational theory, free from any “mysticism incompatible with the true spirit of science” [10] (p. 83) [20] (v. 2, p. 63). By referring to “the assumption of a coupling of quantum processes in separated atoms”, Bohr also captured the core of the dilemma later posed by the EPR experiment. Bohr linked this dilemma to the impossibility of visualization, ultimately making how what is observed in the experiment comes about invisible to thought. What makes Bohr’s above statement remarkable is that it was made in 1925, 10 years before EPR’s article [11]. It is true that Einstein brought up related considerations in his exchanges with Bohr already in 1927, which was still two years away in 1925 [20] (v. 2, pp. 41–58).

There are numerous invocations of the limits of visualization throughout Bohr’s writing, with an increasing emphasis, ultimately amounting to dealing with the invisible to thought, even without using this language (e.g., [20] (v. 1, pp. 52, 77, 98, 108, 114–115, 118, v. 2, p. 51, v. 3, p. 25) [10] (p. 88, 152). I have considered this aspect of Bohr’s argumentation in [2] (pp. 227–257) [26] (pp. 38–40, 63–64, 170–173) [27] (pp. 16–17). I would like, however, to discuss Bohr’s 1949 comment on the EPR experiment: “Certainly the issue [raised by the EPR experiment] is of a very subtle character and suited to emphasize how far, in quantum theory, we are *beyond the reach of pictorial visualization*” [20] (v. 2, p. 59; emphasis added). As Bohr’s 1925 statements cited above make clear, for Bohr, the cost of “the assumption of a coupling of the processes in separated atoms”, at stake in the EPR experiment (which deals with two spatially separated quantum objects) was the impossibility of “the ordinary spacetime description” of how phenomena observed there are possible. The EPR experiment and, in fact, all quantum experiments “preclude the possibility of a simple description of the physical occurrences [i.e., of how of phenomena considered comes about] *by means of visualizable pictures*” [25] (v. 5, p. 311, Letter to Born, 1 May 1925). EPR becomes equal to RWR.

Bohr, again, assumed that such may be the case well before EPR’s article, which might be one of the reasons why he thought that EPR’s experiment did not contain anything essentially new. He was not entirely right in this assessment, given the role of entanglement and correlations brought about by the EPR experiment. EPR, however, did not consider these concepts, nor did Bohr in his reply. Entanglement was introduced by Schrödinger in response to EPR’s paper [1,16]. Correlations became prominent even later. In any event, Bohr argued that, while confirming “how far, in quantum theory, we are *beyond the reach of pictorial visualization*”, EPR’s argument did not demonstrate that QM is either incomplete or else nonlocal, in the sense of allowing an instantaneous action at a distance. QM allows for other concepts of non-locality. I have considered Bohr’s argument in detail in [2] (pp. 227–252). My main point here is Bohr’s view that the ultimate reality responsible for quantum phenomena is beyond visualization and ultimately is invisible to thought, while quantum phenomena are visible to thought and are, in fact, available to our immediate phenomenal perception, and as such can be treated by CP.

It might be useful to consider, as a simple representative example, how predicting the polarization of a photon appears in RWR interpretations. There are two possible outcomes of measurement (after the initial preparation): for example, the horizontal state *x* and the vertical state y, either observed classically by the Bohr postulate. In RWR interpretations, one could not say, as is common, that before it is measured, the photon is in a superposition of two physical states. In the present view, moreover, the concept of photon, while it cannot be observed as such (only the corresponding effect in measuring instruments can) is only applicable at the time of observation by the Dirac postulate. The wave function, a vector in the Hilbert space (over C), associated with the system by QM, allows one to predict that either physical state *x* or *y* is written as $\left|\left.\psi \right\rangle =\alpha \right|\left.X\right\rangle +\beta \left.|Y\right\rangle$ with the probability amplitude of |$\left.\psi \right\rangle$ associated with state vector $\left.|X\right\rangle$ given by α and $\left.|Y\right\rangle$ given by β. The probability amplitude, usually associated with and, in fact, defined by the wave function, or reciprocally defining the wave function, provides the probability density by the square modulus of |$\left.\psi \right\rangle$, ${\left\|\psi \right\|}^{2}$. The latter is always a positive real number and can be normalized so the probability is between zero and one. In a random experiment, the probability of the photon when its polarization will be measured, being horizontally polarized is $\;{\left|\alpha \right|}^{2}$, and being vertically polarized is ${\left|\beta \right|}^{2}$ (by Born’s rule). That, however, in RWR interpretations, does not mean that $\left|\left.\psi \right\rangle =\alpha \right|\left.X\right\rangle +\beta \left.|Y\right\rangle$ represents the photon in a superposition of two physical states, *x* and *y*, as nothing can be said concerning what happens between observations in RWR interpretations. Only the mathematical vectors, designated as $\left.|X\right\rangle$ and $\left.|Y\right\rangle$ (in capital letters), in the Hilbert space used, are in a linear (mathematical) superposition, with given amplitudes, and not quantum objects, let alone the outcomes of experiments. Actual predictions will involve *h*, which does not appear in these abstract notations but is necessary for predicting these outcomes.

Quantum predictions also require rules added to the formalism, such as Born’s rule, which relates complex quantities of the formalism to real numbers corresponding to the probabilities of quantum events. While there are other rules that have the same function, I shall only refer to Born’s rule (the first and still most commonly used). It is worth restating how Born’s rule works with RWR interpretations and under the assumption of the Bohr postulate. One takes the square moduli of the eigenvalues of the operators associated with quantum variables, such as position, momentum, or energy, or equivalently, multiply these eigenvalues by their complex conjugates. Doing so gives one real number, corresponding, suitably normalized, to the probability densities of observable events. Thus, when ψ is a wave function for a point particle in position space, the probability density function *p (x*, *y*, *z)* for predicting a measurement of the position at time *t*_1_ equals to ${\left|\psi (x,y,z,t_{1}\right|}^{2}$. Integrating over this density gives the probability or (if one repeats the experiment many times) statistics of finding the particle in a given area. Born’s rule is a purely mathematical procedure, which has no representational relation to physical reality any more than does the formalism itself. The only such relations are those to quantum phenomena observed in measuring instruments and represented (along with the observable parts of these instruments) by the Bohr postulate and by the formalism of CP, defined over R, and not that of QM, defined over C.

Born’s rule brings physics into the purely abstract mathematics of QM or QFT. In fact, it establishes a definition of the wave function in its relation to physics in RWR and most interpretations. Born’s rule is quantum, but it connects QM to the observable outcomes of experiments, which outcomes are classical. Originally, Schrödinger intended his wave function to represent, in wave terms, the ultimate reality responsible for quantum phenomena. His hope of achieving this goal became more difficult and was abandoned by him soon after he introduced his time-dependent equation, which requires a complex wave function, initially defined as a real entity [2] (pp. 145–166). Attempts at realist interpretations of the wave function have never stopped, and Schrödinger returned to such an attempt in the 1950s [28]. Although Born’s or similar rules are connected naturally to the formalism of QM (given that complex conjugation naturally connects complex and real numbers), they are added to rather than contained in this formalism. We do not know why these rules work. But then, we do not know why the whole scheme of QM works either, at least in RWR interpretations where it does not represent either the ultimate reality responsible for quantum phenomena or the quantum phenomena themselves, represented by CP.

As stated from the outset, Bohr eventually adopted the term “phenomenon” to refer only to what is observed in measuring instruments and grounded his ultimate interpretation in this concept:

I advocated the application of the word phenomenon exclusively to refer to *the observations obtained under specified circumstances*, including an account of the whole experimental arrangement. In such terminology, the observational problem is free of any special intricacy since, in actual experiments, all observations are expressed by unambiguous statements referring, for instance, to the registration of the point at which an electron arrives at a photographic plate. Moreover, speaking in such a way is just suited to emphasize that the appropriate physical interpretation of the symbolic quantum-mechanical formalism amounts only to predictions, of determinate or statistical character, pertaining to individual phenomena appearing under conditions defined by classical physical concepts [describing the observable parts of measuring instruments]. [20] (v. 2, p. 64; emphasis added)

Rigorously, one can no longer speak of the electron having arrived at a photographic plate, which implies a classical-like motion. Instead, the spot on the plate registers, in the corresponding phenomenon, the electron, as the quantum object considered. This is, however, clearly what Bohr means, given his overall argument in this article. The difference between phenomena and objects has its genealogy, in modern times in Kant’s philosophy. As noted, Kant distinguishes between objects as things-in-themselves, as existing independently, and phenomena, as entities created by our mind, which may not correspond to the objects in nature (the main concern in physics) they are aiming to represent [24]. In RWR interpretations, quantum phenomena are not related to quantum objects at all but, if one assumes the Bohr postulate, represent classical physical objects observed in quantum experiments, such as a spot on a photographic plate. Strong RWR interpretations are more radical than Kant’s epistemology, developed of course with Newton’s physics in mind. While Kant’s things-in-themselves were assumed to be beyond knowledge, they were not assumed to be beyond conception, at least a hypothetical conception, even if such a conception could only be practically justified by its applications [24] (p. 115). By contrast, in strong RWR interpretations, what is practically justified is not a possible conception of the ultimate reality responsible for quantum phenomena, but the impossibility of such a conception [2] (pp. 57–58).

As defined by “the observations [already] obtained under specified circumstances”, phenomena refer to events that have already occurred and not to possible future events, such as those predicted by QM. This is the case even if these predictions are ideally exact, which they can be in certain circumstances, such as those of EPR-type experiments. The reason that such a prediction cannot define a quantum phenomenon is as follows. A prediction for variable *Q* (for example, one related to a coordinate, *q*) cannot, in general, be assumed to be confirmed by a future measurement, in the way they can be in CP or relativity, where all measurable quantities are always defined simultaneously and assume to have a reality independent of measurement. In QP, one can always, instead of the predicted measurement, perform a complementary measurement, that of *p* (the momentum), which will leave any value predicted by using *Q* entirely undetermined by the uncertainty relations. This measurement would, in principle, preclude associating a physical reality corresponding to a coordinate *q* when one measures *p* [2] (pp. 210–212). (This point has major implications for understanding the EPR experiment and countering EPR’s argument along the lines of Bohr’s reply [2] (pp. 227–257)). One can never speak of both variables unambiguously, even if they are associated with measuring instruments. Any reference, even that of a single property of a quantum object, is impossible in RWR interpretations, even at the time of observation. This is why classical causality does not apply in the way it does in CP, even when probability is used there, or relativity (which is a deterministic theory, in which all predictions are ideally exact). In CP, this difficulty does not arise because one can, in principle, always define both variables simultaneously as independent from observation for all individual processes, including those underlying the behavior of complex systems treated probabilistically. As a result, one can always unambiguously speak of the reality associated with both variables and assign them to the object itself. By contrast, in a quantum experiment, one always deals physically with a system containing an object and an instrument. Thus, in considering quantum phenomena, there is, on the one hand, always discrimination between an object and an instrument, and, on the other, the impossibility of separating them. This impossibility compelled Bohr to speak of “the essential ambiguity involved in a reference to physical attributes of objects when dealing with phenomena where no sharp distinction can be made between the behavior of the objects themselves and their interaction with the measuring instruments” [20] (v. 2, p. 61). By contrast, a reference to what is observed can, as classical by the Bohr postulate, be unambiguous and unambiguously communicable.

There are several reasons for adding the Dirac postulate to the Heisenberg and Bohr postulates, both in QM and even more so in QFT, as explained above and considered in [2] (pp. 273–306) and [3,4,5], beginning with the fact that no properties can be assigned to a quantum object apart from observations in strong RWR interpretations. In any strong RWR interpretation, nothing at all can be said or even thought about what happens between observations. Need one, then, still speak of quantum objects between observations? Also, as considered in connection with the idea of the so-called “cut” in Section 4, the following situation obtains. On the one hand, in each experimental arrangement defining an observation one must, regardless of interpretation, discriminate “between those parts of the physical system considered which are to be treated as measuring instruments and those which constitute the objects under investigation” [11] (p. 701). On the other hand, the difference between them is not uniquely defined. It is how one sets up an experiment that defines what is “the object under investigation” in this experiment, which invites assuming the concept of a quantum object to be applicable only at the time of observation.

The situation has additional complexity because, while still treatable by means of QM, “the object under investigation” in a quantum experiment may not be a quantum object, defined here as an object that can always be treated by quantum means, when considered by itself. “The object under investigation” by quantum–theoretical means may be partly classical, for example, containing the cat in the cat experiment, which is why Bohr speaks here of “objects under investigation” rather than quantum objects. This object, however, can only be partly classical because it must also contain a properly quantum object, such as an electron or a photon, or some composite quantum object, to observe quantum effects. I shall return to this point, which bears on the cat experiment, in Section 4. I shall merely note for now that the classical part, such as the cat, of such a combined object is always the same object and, hence, does not obey the Dirac postulate, which only applies to quantum objects. In accordance with Bohr’s concept of phenomenon, whatever the object of investigation is in a quantum experiment, it cannot be considered independently of its interaction with the observational instrument used. This impossibility makes any quantum experiment and QT involve both a combination and a separation of an object and an instrument. In the present, strong RWR, interpretation, even though a quantum object is only defined at the time of observation by the Dirac postulate, it is an entity beyond representation or even conception.

As indicated earlier, the Bohr postulate reflects the transition from the classical level of observation to the ultimate (“quantum”) reality by the initial preparation and conversely, from the ultimate (“quantum”) reality to the classical level of observation in a subsequent experiment, testing a possible prediction on the basis of this preparation. (I borrow the language of “preparation” and “observation” from [17] but modify both terms because “observation” can be used more generally, and a “preparation” is also, physically, an observation and then a measurement). According to Bohr:

The essential lesson of the analysis of measurements in quantum theory is thus the emphasis on the necessity, in the account of the phenomena, of taking the whole experimental arrangement into consideration, in complete conformity with the fact that all unambiguous interpretation of the quantum mechanical formalism involves the fixation of the external conditions, defining the initial state of the atomic system concerned and the character of the possible predictions as regards subsequent observable properties of that system. Any measurement in quantum theory can in fact only refer either to a fixation of the initial state [an initial preparation] or to the test of such predictions [predicted observations], and it is first the combination of measurements of both kinds which constitutes a well-defined phenomenon. [10] (p. 101)

Notably, Bohr speaks, along the lines stressed here, of quantum measurements as related to *states* of quantum systems (states only manifested in the classically measured properties of the instruments) and not of observing or measuring the motions of quantum objects. One begins an experiment by classically preparing the instrument used and registering, at time *t_prep_*, the data obtained by the interaction between this instrument and a quantum object. Doing so sets up the future workings of the ultimate reality considered, placed beyond representation or even conception, in RWR interpretations. This measurement is a transition from the classical to the quantum reality—from the visible to thought to the invisible to thought—in this experiment. Such data belong to the observed quantum phenomena and, thus, are independent of any theory. One, however, needs a theory, such as QM, to predict, by using these data, the data obtained in any future experiment. By the same token, the role of CP, defined by the Bohr postulate, acquires its mathematical meaning in the formalism of QT, which is as follows.

One can, for example, use Schrödinger’s equation (considering it in one dimension for simplicity):

$$i\hbar \frac{\partial \psi (x,t)}{\partial t}=\left[-\frac{\hbar^{2}}{2m}\;\frac{\partial }{\partial x^{2}}+V\;(x,t)\right]\psi \;(x,\;t).$$

for making a prediction concerning a future coordinate measurement associated with an electron on the basis of a previously performed position measurement, say, *q_prep_*. Here, *m* is the mass of the particle, *V* (*x*,*t*) is the potential that represents the environment of the electron, as part of the initial conditions, and ψ (x, t) is the wave function that assigns a complex quantity to each point *x* and each time *t*. By Born’s rule, the wave function is associated with the probability amplitude, in fact defining the wave function, by providing the probability density by the square modulus of |$\left.\psi \right\rangle$, ${\left\|\psi \right\|}^{2}$, which is always positive and can be normalized so the probability is always between zero and one. Then, to confirm such a prediction, one sets up a suitable observational device and makes a new observation at time *t_test_* registering an outcome, *q_test_*, of the experiment, thus predicted. The instrument needs to be prepared in accordance with this prediction for the coordinate measurement. This is because, as explained, one can always perform a different type of measurement, say, that of the momentum, at *t_test_*, which will irrevocably disable verifying the prediction concerning *q*. This is the transition from the “quantum” to the “classical” reality in this experiment—from the invisible to thought to the visible to thought—in this experiment. One cannot associate any concept of “quantum” with the ultimate reality responsible for quantum phenomena this reality in RWR interpretations. I speak of this reality as “quantum” because CP, which describes the data thus classically observed in two experiments, cannot predict them. (The addition of SR in high-energy quantum regimes does not affect the situation, as one still needs QFT for making such predictions). Mathematically, the classical to “quantum” transition means putting the data, say, the measured value of the coordinate and time *t_prep_* into Schrödinger’s equation above. One then can, adding Born’s rule to the solution, predict the probability of the quantum to classical transition. So, the quantum to classical transition is the use of the formalism, cum Born’s rule, and then the measurement to confirm this prediction.

The procedure just considered allows RWR interpretations to establish the connection between the formalism of QM and the data observed in quantum experiments. The nature of this connection is different not only physically or epistemologically, but also mathematically in defining QM (or QFT) as, in Bohr’s terms, a “symbolic theory” (e.g., [20] (v. 1, p. 76; v. 2, p. 64). It is symbolic because, unlike those of classical mechanics or relativity, the mathematical symbols comprising the formalism of QM, do not represent or contain any physical relations, with “what we have done and what we have learned” in quantum experiments themselves. These symbols only allow one to predict, by using in addition to Born’s rule, the probabilities of what can be observed, on the basis of the data observed in measurements previously performed. Explaining what “we have done and what we have learned” in quantum experiments implies that “the account of the experimental arrangement and the results of the observation must be expressed in unambiguous language with suitable application of the terminology of classical physics” [20] (v. 2, p. 39). In other words, it implies the Bohr postulate. This account represents unambiguously communicable experimental “facts” such as the occurrence of spots on a photographic plate in a quantum experiment. In Bohr, or here, the nature of the “contact” between the symbolic formalism of QM and phenomena (and, hence, these classically explainable actual “facts”) is as follows. Classical explanations only refer to these “facts” as phenomena manifested in the observable parts of measuring instruments. These instruments also, necessarily, have quantum strata through which they interact with quantum objects or the ultimate constitution of the reality responsible for quantum phenomena. Both phenomena and the observable parts of measuring instruments are treated classically, but the quantum-mechanical formalism and its symbols do not represent (and, except by Born’s rule, have no connections to) this classical treatment. The symbols of formalism are only used for predicting the data or information thus observed. This information is classical in nature, although the organization or structure of this information, for example, as observed in quantum correlations, cannot be predicted by CP. The symbols of QM, which belong to our thinking, only relate to the probabilities of the outcomes of quantum experiments, with Born’s rule, added, as a separate postulate, to the formalism. Born’s rule, again, provides the only connection, probabilistic in nature between these symbols and the observed reality, thus also connecting the mathematics of QM over C to the data in space and time, represented by the mathematics over R. Accordingly, these symbols do not in any way represent or physically relate, otherwise than via Born’s rule, to what is observed in quantum experiments. While, as with any mathematical symbols, these symbols are also unambiguously communicable, they are not related to the outcomes of measurements, which are communicated “in terms of in unambiguous language with suitable application of the terminology of classical physics”. Only Born’s rule relates to these two types of entities.

As Bohr stated in his reply to EPR: “In accordance with this situation [that at stake in QM] there can be no question of any unambiguous interpretation of the symbols of quantum mechanics other than that embodied in the well-known rules which allow us to *predict* the results to be obtained by a given experimental arrangement described in a totally classical way” [11] (p. 701; emphasis added). Obviously, given their strictly predictive roles, these symbols—“embodied in the well-known rules which allow us to predict [these] results” but not in these classically-described results themselves—in no way represent the classical and in part daily-language or narrative accounts of “what we have done and what we have learned” [20] (v. 2, p. 39). These accounts, again, only pertain to the outcome of quantum experiments, and they can be communicated unambiguously, as can, again, be the formalism of QM. As discussed below, this unambiguous communication is the meaning of objectivity for Bohr [20] (v. 2, pp. 68–69, v. 3, p. 7). QM or QFT is a theory of the future, and not of the present or the past, defined by the archive established by already performed measurements, independent of QM or QFT, which only deals with predictions of possible new entries into this archive.

The question might be asked in connection with the Dirac postulate: If a quantum object is only an idealization defined by an observation, rather than something that exists independently (as the ultimate reality responsible for quantum phenomena is assumed to exist), could one still speak of the same quantum object, say, the same electron, in two successive observations, as just considered? The case can be given an RWR interpretation, as all these properties are, physically, those of measuring devices, impacted by quantum objects, rather than of these objects themselves, placed beyond representation or conception. Rigorously speaking, if the concept of a quantum object is only applicable at the time of observation, then a prediction based on a given measurement and the new measurement based on this prediction could only concern a new quantum object. It cannot concern the object that one used in making the first measurement. One deals with two different quantum objects, two different electrons, for example. To consider them as the same electron is, however, a permissible idealization in low-energy QM, or low-energy QFT, regimes. This idealization is still statistical because there is a probability (in general low) that the second observation will register nothing or will register a different electron. By contrast, speaking of the same electron in successive measurements in high-energy (QFT) regimes is meaningless, because they can register quantum objects of different types, say, in the case of QED an electron in the initial and a positron or photon in the next measurement [2] (pp. 279–292) [3].

On the other hand, there is no difficulty in speaking of the same classical object, even if it is part of the object of investigation by quantum means, such as QM, in a quantum experiment, which, again, requires a ‘properly quantum object’, such as an emitted particle in the cat experiment, to be a quantum experiment. Thus, at all stages of the cat experiment, we deal with the same cat, dead or alive. The state of a classical object can change in time; the state of an observational instrument usually does when impacted by a quantum object. As Heraclitus said, one cannot step into the same river twice because neither the river nor the one who steps into it is the same. Nevertheless, classical or relativistic objects themselves can be considered the same, in their different states, for all practical purposes, and strictly distinguished from each other. One always deals with an idealization that is visible to thought or, in some cases, observable independently of measuring instruments. The case is very different in considering quantum objects. First, while quantum objects can change their location, momentum, or energy, that is, the measurement associated with a variable can be different, quantum objects are considered indistinguishable from each other in terms of such invariant characteristics as mass, charge, or spin. Secondly, in RWR interpretations, even these quantities are only observable as effects of the interactions between quantum objects and observational instruments. This is because no concepts apply to quantum objects, whether one defines them as existing independently or, as here, only at the time of measurement. In short, quantum objects are always invisible vs. the traces left by their interactions with observational instruments. In strong RWR interpretations, moreover, they are invisible to thought in the sense of being beyond thought’s capacity to *think* them.

Two key concepts defining CP and relativity, measurement, and classical causality, become no longer applicable in QT in the present view, or all RWR interpretations that assume the Bohr postulate. I discuss measurement first. The classical concept of measurement belongs to CP and the history that shaped it, beginning with ancient Greek thinking and the rise of geometry, geo-*metry*, arguably the first science of measurement. In the present view, what is commonly referred to as a quantum measurement does not measure, or in the first place, is not an observation of, any property that the ultimate reality responsible for quantum phenomena would be assumed to possess before or even during the act of observation. The concept of observation requires a redefinition as well. An act of observation in QP establishes, creates, quantum phenomena through an interaction between the instrument and the quantum object. This act is a unique event of creation. While this formulation echoes J. A. Wheeler’s appeal, inspired by Bohr, to “an elementary act of creation”, the present view or that of Bohr’s is different from Wheeler’s view. This is because Wheeler’s view is based on the concept of a “participatory universe”, never invoked or, I would argue, found in Bohr [29,30] (pp. 189, 194) [2] (pp. 168–169, 220–221). As existing independently of us, as it is assumed to be in the present and Bohr’s view, (the reality of) the universe is not participatory, although it can be—and in QP is—changed by our experiments, but only by assuming the locality principle. This principle states that physical systems can only be physically influenced by their immediate environment. (Relativity specifically prohibits the transmission of such influences faster than *c*). Importantly, RWR interpretations do not entail a uniform character of the ultimate reality considered, while manifesting itself differently in each quantum experiment (e.g., [31] and, qualifying this view, [32]). This assumption is precluded by strong RWR interpretations, which disallow any conception of this reality and, hence, that of its unity or oneness. While each time unthinkable, the ultimate reality responsible for quantum phenomena is each time unique, manifesting its uniqueness in each encounter with it through its effect (or set of effects).

This view gives a central significance to the category of event, as defining a new physical situation each time, akin to important, “revolutionary” events that transform the situation in life or culture. In QP, however, every event of observation radically transforms the situation and redefines the possible future, making the preceding events no longer meaningful for predictions concerning future events from this point on. (In particular, as defined by a classically described effect of RWR on experimental technology, the concept of the events thus assumed are different from other event-based interpretations of QM or QFT, especially realist ones, such as by R. Haag [33,34], who presents a prominent earlier example, and several recent works (e.g., for a strong realist view, in the absence of a classical description of observation [35]). QT becomes a theory of transition probabilities between events, each defined by experimental technology and our decisions concerning which experiment to perform. Then what is observed as the data can be measured classically, just as one measures what is observed in CP. In CP, however, what is observed or measured could be associated with the object considered. In QP, there is always a difference between observations, which construct phenomena, and measurements, which classically measure the physical properties of physical entities (classical objects) represented by phenomena.

It follows, that measuring instruments contain both an observable and, thus, visible (even to an immediate perception) classical stratum and a quantum stratum, which enables their interactions with quantum objects. This stratum and, as quantum, this interaction cannot be observed and, in RWR interpretations, is invisible to thought. This interaction is, in Bohr’s language, “irreversibly amplified” to the classical level of observable effects, such as a spot left on a silver screen [20] (v. 2, p. 73). The physical nature of this “amplification” is part of the problem of the physical emergence of classical reality from quantum reality or, in present terms, the reality ultimately responsible for quantum phenomena. The problem is, commonly, including by this author, seen as unsolved. There are arguments to the contrary, for example, on lines of decoherence or consistent histories approaches, quite prominent, especially those for decoherence, in recent discussions of quantum foundations (e.g., [36] for a comprehensive overview and further references). These claims have been challenged and are not generally accepted. Fortunately, quantum phenomena and QM allow us to bypass this problem in quantum observation. As Bohr noted in 1937, QM is “justified only by the possibility of disregarding in its domain of application the atomic structure of measuring instruments themselves in the interpretation of the results of experiments” [10] (p. 88). This disregard, Bohr added, may lead to new complexities in high-energy regimes [10] (p. 88). “The atomic structure of measuring instruments” is still disregarded in high-energy regimes and QFT, which may be responsible for the appearance of infinities and the necessity of renormalization. These complexities have not always been seen as adequately resolved, notwithstanding the predictive capacity of QFT, in part enabled by renormalization. QED is the best confirmed physical theory ever.

The nature of causality in QM or QFT changes as well, as classical causality is no longer possible in RWR interpretations, or certain other interpretations [2] (pp. 207–218). By “classical causality”, I refer to the claim that the state, *X*, of a physical system is determined, in accordance with the law, at all future moments of time once its state, *A*, is determined at a given moment of time, and state *A* is determined by the same law by any of the system’s previous states. This assumption implies a concept of reality, which defines this law, thus making this concept of causality ontological. There are several reasons for my choice of “classical causality”, rather than just causality, commonly used to designate this type of concept. (This term is sometimes used differently, for example, in the context of Bell’s theorem). The main one is that it is possible to introduce alternative, probabilistic, concepts of causality, applicable in QM, including in RWR interpretations, where classical causality does not apply (e.g., [2] (pp. 207–218)). In [2] the probabilistic concept of quantum causality is defined as follows: “An *actual* event that has happened determines which events *may or (in view of complementarity) may not* happen and be predicted with *one probability or another*, which is not the same that any of them *will* happen. This event, A, at time *t*_0_ defines certain *possible*, but *only possible* future events, say, X, at time *t*_1_” [2] (pp. 208–209).

Some, beginning with P. S. Laplace, have used “determinism” to designate classical causality. I use “determinism” as an epistemological category referring to the possibility of predicting the outcomes of classically causal processes ideally exactly. In classical mechanics, when dealing with individual or small systems, both concepts are co-extensive. On the other hand, classical statistical mechanics or chaos theory are classically causal but not deterministic in view of the complexity of the systems considered, which limits us to probabilistic or statistical predictions concerning their behavior.

In dealing with quantum phenomena, deterministic predictions are not possible even in considering the most elementary quantum systems. This is because, as noted, the repetition of identically prepared quantum experiments in general leads to different recorded data, and unlike in CP, this difference cannot be diminished beyond the limit, defined by *h*, by improving the capacity of our measuring instruments. These data are both recorded in the initial measurement (the initial preparation), enabling a prediction, and recorded in the second measurement (the predicted observation), which would verify this prediction. These recordings will be different either when one repeats the whole procedure in the same set of experimental arrangements or when one builds a copy of the apparatus and sets it up in the same way, as we do to separately verify the outcomes of experiments. Either repetition is possible because the preparations of the instruments could be controlled classically. On the other hand, their interaction with quantum objects (or by the Dirac postulate, the ultimate reality responsible for quantum phenomena) cannot be controlled. Bohr speaks of “the finite and uncontrollable interaction between the objects and the measuring instruments in the field of quantum theory” [12] (p. 700). The most crucial, however, is the difference in the outcomes of the second measurement in repeated setups. One can prepare any given state, as manifested in the corresponding measurement, even though one cannot do so in a single preparation but only by post-selecting the required preparation. By contrast, the outcome of the second (predicted) measurement cannot be controlled, only allowing one to predict its probability. There are situations, such as those of the EPR-type experiments, where exact predictions are ideally possible, but with important qualifications, which still preclude classical causality [2] (pp. 203–216).

It follows that the probabilistic or statistical character of quantum predictions must, on experimental grounds, hold in interpretations of QM or alternative theories of quantum phenomena (such as Bohmian mechanics) that are classically causal. QM or QFT, in RWR interpretations, are not classically causal because the ultimate nature of reality responsible for quantum phenomena is assumed to be beyond a representation or even conception. Classical causality would imply at least a partial conception and even representation of this reality. These circumstances imply a different reason for the recourse to probability or statistics in QT in RWR interpretations, emphasized by Bohr:

[I]t is most important to realize that the recourse to probability laws under such circumstances is essentially different in aim from the familiar application of statistical considerations as practical means of accounting for the properties of mechanical systems of great structural complexity. In fact, in quantum physics we are presented not with intricacies of this kind, but with the inability of the classical frame of concepts to comprise the peculiar feature of indivisibility, or “individuality”, characterizing the elementary processes. [20] (v. 2, p. 34)

The “indivisibility” refers to the indivisibility of phenomena in Bohr’s sense, as the impossibility of considering quantum objects independently of their interactions with these instruments. “Individuality” refers to the fact that, as the outcome of a unique act of creation, each phenomenon is individual and unrepeatable, as well as discrete relative to any other phenomenon. This individuality also embodies the essential randomness contained in quantum phenomena. This randomness is not found in CP, because even when one must use probability there, at the bottom, one deals with individual processes that are classically causal. Hence, randomness does not ultimately exist or is assumed ultimately not to exist. In principle, one can isolate an individual constituent of a complex mechanical system, say, a molecule of gas, and predict its behavior ideally exactly. This is not possible in considering individual quantum systems, no matter how elementary. By the same token, in strong RWR interpretations, such systems or the ultimate nature of the reality considered can never be made available to thought and, hence, it cannot be assumed to be classically causal either, which would make them available to thought, at least partially.

QP, however, only contains essential randomness, rather than is entirely random, because it allows for probabilistic or statistical predictions (purely random events do not) and, more crucially, correlations, such as EPR-type correlations, at stake in Bell’s or the Kochen–Specker theorem. One of the greatest mysteries of QP is how random individual events can, under certain circumstances, give rise to an order, not only a statistical correlational order but an order nonetheless [2] (pp. 253–256). QM predicts these correlations, but RWR interpretations do not explain them any more than they explain how any single outcome involved comes about. (I put aside the question of statistical vs. probabilistic interpretations of QM, considered from the RWR perspective in [28] (pp. 173–186) [37]. A compelling realist statistical interpretation of QM that, nevertheless, has affinities with the present interpretation is offered in [2,38] (p. 62, n. 20)).

I shall now explain Bohr’s concept of complementarity, specifically as it appears in his ultimate interpretation of quantum phenomena, where it applies to phenomena in Bohr’s sense. As defined more generally, complementarity is characterized by the following:

(A) A mutual exclusivity of certain phenomena, entities, or conceptions; and yet
(B) The possibility of considering each one of them separately at any given point; and
(C) The necessity of considering all of them at different moments for a comprehensive account of the totality of phenomena that one must consider in QP.

Complementarity, thus, implies two incompatible pictures of what is observed, as phenomena, in the instruments used. The *possible* information about a quantum object, the information *to be found* in observational instruments, is only obtainable in the mutually incompatible evidence under different experimental conditions [20] (v. 2, p. 40). On the other hand, once made, either measurement, say, that of the position, will provide the complete *actual* information about the system’s state, as complete as possible, at this moment in time. One could never obtain the complementary information provided by the momentum measurement, at this moment in time, because to do so one would need simultaneously to perform a complementarity experiment on it, which is impossible. But one can, by (B), always decide to perform either one or the other experiment. Each experiment establishes the only reality there is at this moment in time, and the alternative decision would establish a different reality. Thus, rather than arbitrarily selecting one or another part of a preexisting physical reality, as in CP, our decisions concerning which experiment to perform establish the single reality that defines what type of quantity can be observed or (probabilistically) predicted and precludes the complementary alternative.

Bohr’s complementarity is, thus, not only about a mutual exclusivity of things, necessary as it is in QP, but also about performing quantum experiments by human agents, in which this mutual exclusivity is defined by the agents’ decision concerning which experiment to perform. One can set up a device to perform an experiment and treat a natural event as an experiment, but any such setup is only possible by a human agent, individual or collective. It is this decision-making aspect of complementarity, as defining the reality considered and the future course of this reality, and its implications discussed below that are especially important for this article’s argument. This argument is less concerned with the mutual exclusivity of complementary entities (point (A) above), more commonly focused on, often at the expense of other aspects of Bohr’s concepts, thus misunderstanding it. As Bohr noted, the ability to freely or sufficiently freely make a decision (a preferable category to “choice”) concerning which experiment one wants to perform is in accordance with the very idea of experimentation in all physics or science [12] (p. 699). Contrary to the case of CP or relativity, however, in QP implementing our decision concerning what we want to do will allow us to make only certain types of predictions and will irrevocably exclude certain other, complementary, types of possible predictions. Because of the complementarity, this decision defines the reality considered and its possible (but only possible given the irreducible role of probability in QT) future course in two alternative ways. There is only one such course in CP or relativity. By the same token, in these theories our decisions, even if they define a new experimental situation, would then allow us merely to follow what happens in any event, without interfering with the phenomena observed. CP or relativity always allows us to determine, as independent of observation, all variables necessary for defining the future course of reality, in accord with classical causality. This is never possible in QP because of the uncertainty relations and complementarity, even if one assumes the underlying classical causality, as in certain interpretations of QM (such as many-worlds ones), or in Bohmian mechanics, which contains both the uncertainty relations and complementarity. Both, it is worth emphasizing, are features of quantum phenomena, rather than QM, to which, as noted from the outset, an RWR interpretation may apply independently of QM. On the other hand, Bohmian mechanics, which is a different theory of quantum phenomena and, as such, must and does relate to complementarity (or the uncertainty relation), is a realist and classically causal theory in all interpretations. In any event, in QP we always have the freedom, at least a sufficient degree of freedom, to make this decision or to change it and, thus, to define a future course of reality. It is true that this freedom is denied by so-called “superdeterminism”, according to which all our decisions are pre-determined in advance, from the Big Bang, on. This view, controversial in any event, is put aside here as, by definition, realist.

It is worth noting that wave-particle complementarity, with which the concept of complementarity is often associated, had not played a significant, if any, role in Bohr’s thinking, especially after the Como lecture introducing the concept of complementarity, defined somewhat differently and less sharply than in his later works [20] (v. 1, pp. 54–55). Bohr was always aware of the difficulties of applying the concept of physical waves to quantum objects or assuming that both types of behavior, particle-like and wave-like, pertain to the same individual entities, such as each photon or electron. Bohr’s solution to the dilemma of whether quantum objects are particles or waves was that they were neither, any more than anything else, by the Heisenberg postulate. Instead, either “picture” refers to one of the two mutually exclusive sets of discrete individual effects, described classically by the Bohr postulate, of the interactions between quantum objects and measuring instruments, particle-*like*, which may be individual or collective, or wave-*like*, which are always collective, composed of discrete individual effects. Examples of the latter are interference effects, composed of a large number of discrete traces of the collisions between the quantum objects and the screen in the double-slit experiment in the corresponding setup (when both slits are open and there are no means to know through which slit each object has passed). These two sets of effects may be seen as complementary: they are mutually exclusive and require mutually exclusive experimental setups to be observed. In CP, wave and particle phenomena or (they can be identified) objects are treated by *two mutually exclusive* theories. This is, however, not the same as being complementary in Bohr’s sense. The latter must include the (B) and the (C) part of the concept, applicable to the same object but leading to different phenomena by (A), depending on which setup one decided to use, predicted by *the same theory*, QM or QFT, but predicted differently in each setup. This is not the case in CP where all variables considered by a given theory, or either wave or particle type, can, in principle, always be simultaneously defined exactly (there are no uncertainty relations), which also ensures classical causality.

Accordingly, in QP one always deals with incompatible observable physical effects in complementary contexts. On the other hand, the mathematical formalism of QM offers correct probabilistic or statistical predictions of quantum phenomena *in all contexts*, in RWR interpretations under the assumption that the ultimate nature of reality responsible for quantum phenomena is invisible to thought. This situation is also responsible for what is known as “contextuality”, considered from the RWR perspective in [39]. (A helpful survey of contextuality is offered in [40]). As Bohr observed:

Just in this last respect [of the renunciation in each experimental arrangement of the one or the other of two aspects of the description of the physical phenomena] any comparison between quantum mechanics and ordinary statistical mechanics,—however useful it may be for the formal presentation of the theory,—is essentially irrelevant. Indeed we have in each experimental arrangement suited for the study of proper quantum phenomena not merely to do with an ignorance of the value of certain physical quantities, but with the impossibility of defining these quantities in an unambiguous way. [12] (p. 699)

Complementarity, thus, reflects the fact that, bringing together two main meanings of the word “experiment” (as a test and as innovative creation), the practice of QP became the first practice of physics or science that is both, and jointly, *generatively* experimental and *generatively* mathematical. It is generatively experimental as experimental physics in its conventional sense because it is no longer defined, as in CP or relativity, in tracking the independent behavior of the systems considered and measuring their independent properties. It is defined by *unavoidably* creating configurations of experimental technology containing traces of its interactions with nature. Correlatively, what happens is *unavoidably* defined by what kinds of experiments we perform, and by how we affect physical reality by a unique act of observation as creation. I qualify (twice!) by “unavoidably” because while the phenomena observed in CP or relativity may be affected by experimental technology, in principle, one can observe these phenomena, including those staged by new experiments, without affecting what is observed. This also allows one to see these phenomena as ideally representing the corresponding objects. One still follows what happens regardless of this following. In QP, an experiment is a creation of a new material configuration, discrete relative to any other such configuration, thus giving the corresponding meaning to quantum discreteness. This discreteness as such does not preclude the possibility that quantum phenomena in related experiments are continuously connected. This possibility is, however, in principle, precluded in (strong) RWR interpretations, in which how quantum events come about is beyond the reach of thought and, thus, cannot be assumed to be either continuous or discrete.

Correlatively, the practice of theoretical physics in QT is generatively mathematical because it no longer consists, as in CT or relativity, in offering an idealized mathematical representation of quantum objects and behavior. One does not proceed from a physical concept to its mathematization, however partial. The practice of theoretical physics consists in inventing abstract mathematical schemes enabling us to predict, probabilistically or statistically, the outcomes of quantum experiments. These schemes are unrelated to and, thus, are not helped by our general phenomenal intuition of objects and motions, as are those of CP or, in a limited way, relativity. On the other hand, they are not limited by this intuition either.

In fact, one experiments with mathematics as well. It involves experimenting with abstract mathematics, divorced from our phenomenal intuition or even physical concepts; this approach, which began with Heisenberg, led the way to new theories in fundamental physics. Dirac’s work in QED was an even more striking example of this new approach to theoretical physics, which he indeed expressly advocated as “the most powerful method of advance that can be suggested at present” [41] (p. 1). Heisenberg was the first practitioner of this method. To arrive at QM, he abandoned the ways in which mathematics was used in CP or relativity. His method was defined by finding the mathematical scheme that only probabilistically predicted the data considered, without (thus, assuming a weak RWR interpretation) considering how these data came about.

## 3. The Bohr–Schrödinger Exchange on Classical Concepts in Quantum Measurements

Bohr’s view of the unavoidable role of classical physical concepts in considering observation instruments and, hence, of CP in QP is central to my argument here, which converts this view into the Bohr postulate. It is instructive to consider Schrödinger’s comments on this aspect of Bohr’s thinking in a letter written by Schrödinger after reading Bohr’s reply to EPR (in a prepublication version), while working on his cat paradox paper, and Bohr’s reply to Schrödinger. The exchange, relevant to Schrödinger’s overall argument in the cat paradox paper, might have affected his view of the cat experiment, as potentially countering Bohr’s argument. The origin of the experiment appears to have been based on a suggestion by Einstein [42]. Schrödinger’s letter and Bohr’s reply resume an earlier exchange from 1928–1929 on the subject among Einstein, Schrödinger, and Bohr [2] (pp. 32–34). Schrödinger writes:

You [Bohr] have repeatedly expressed your definite conviction that measurements must be described in terms of classical concepts. For example, on p. 61 of the volume published by Springer in 1931 [the original German edition of [20], v. 1]: “It lies in the nature of physical observation, that all experience must ultimately be expressed in terms of classical concepts, neglecting the quantum of action” [[20] (v. 1, pp. 94–95)]. And ibid. p. 74 “the invocation of classical ideas, necessitated by the very nature of measurement” [[29] (v. 1, p. 114)]. And once again [in the reply to EPR] you talk about “the indispensable use of classical concepts in the interpretation of all [proper] measurements” [12] (p. 701), where the printed version adds “proper”. True enough, shortly thereafter you say: “The removal of any incompleteness in the present methods of atomic physics … might indeed only be affected by a still more radical departure from the methods of description of classical physics, involving the considerations of the atomic constitution of all measuring instruments, which it has hitherto been possible to disregard in quantum mechanics”.

This might sound as if what was earlier characterized as inherent in the very nature of any physical observation as an “indispensable necessity”, would on the other hand after all just be a, fortunately still permissible, convenient way of conveying information, a way we presumably sometime will be forced to give up. If this were your opinion, then I would gladly agree. However, the subsequent stringent and clear comparison with the theory of relativity makes me doubt whether, in what I just said, I have understood your view correctly. Because, if we considered the theory of relativity as a conceptual edifice in itself, without any relationships to quantum mechanics, we would presumably never be able to renounce the sharp separation between space and time *in any measurement*. Still, it seems possible that in connection with the unavoidable mutual modification of these two theories, *both* would be forced to shake off their classical eggshell—and that *this* is what you mean. [25] (v. 7, p. 505, Letter to Bohr, 13 October)

Schrödinger admits (“it *seems* possible”) that this may not be what Bohr means, and I would argue that it is not. First, in his reply to EPR, which prompted Schrödinger’s letter, Bohr argued that QM is a complete theory within its scope: It is as complete as nature allows our theory of nonrelativistic quantum phenomena to be, as QM correctly predicts everything that can be established as real in low energy quantum regimes. Accordingly, the “incompleteness” in Bohr’s passage cited by Schrödinger does not refer to QM. It refers to the fact that at the time QFT was not yet adequately developed even in the case of QED. (H. Yukawa’s meson theory of nuclear forces was introduced in the same year 1935). QED only worked then to the first order of approximation, beyond which it led to the appearance of infinities, which were only handled by renormalization fifteen years later. The passage in question was removed from Bohr in the published version of his response to the EPR paper [12], as Bohr explained in his reply to Schrödinger: “I have left out the reference to the possible significance of the atomic constitution of all measuring instruments for the solution of the still unexplained difficulties of electron theory [QED]. The reason is that together with Rosenfeld I am just about to finish a paper about a measuring problem in electron theory in which this question will be elucidated somewhat more fully” [25] (v. 7, p. 511, Letter to Schrödinger, 25 October 1935). (This paper was not published and only became available in Bohr’s collected works [25] (v. 7, pp. 195–209)). In any event, as explained below, Bohr noted that these considerations are irrelevant to EPR’s argument, which deals with QM.

Schrödinger was aware that Bohr also referred to the incompleteness of QED and possibly QFT. It is clear, however, from Bohr’s comment just cited and his related elaborations, including in [20] (v. 1, pp. 89–91, 115), to which Schrödinger refers, that Bohr’s point is not that the *observable*, but crucially, *only* observable, parts of measuring instruments should no longer be described by CP in QFT. Speaking of “a still more radical departure [than in QM] from the method of description of classical physics” only refers to a more radical situation in QFT concerning a possible necessity, as against QM, of considering the atomic structure of measuring instruments, along with its observable parts, still described classically. The latter aspect of quantum observation, or in my terms here, the Bohr postulate, would remain in place in QFT in Bohr’s view, for the reasons discussed earlier in this article and explained in Bohr’s reply to Schrödinger. On the other hand, the atomic constitution of measuring instruments, which can be disregarded in QM, may need to be considered in a relativistic QT, such as QED. It was not clear at the time how to do this and is still not clear. We still do not have a QT that does so, and it is not clear whether such a theory is possible. QFT works well in predicting high-energy quantum phenomena in the absence of such an account, which absence may, however, be responsible for some of its difficulties.

Schrödinger appeared to think that Bohr believed that such a theory, “would be forced to shake off their classical eggshells” of the description of observation instruments, possibly even in QM. But this is clearly not what Bohr thought: classical “eggshells” are part of the phenomena, and unlike in CP, if one shakes them off or breaks them one will only create new eggs with eggshells, without ever exposing, making visible, what is inside. One cannot make an omelet out of the eggs of quantum phenomena, only new eggs. Any subdivision of a quantum phenomenon can only result in a new phenomenon or phenomena, with classically described “shells”, without ever exposing quantum objects. Hence, Bohr speaks of closed phenomena or the wholeness (or indivisibility) of quantum phenomena. As he explained later: “The individuality of the typical quantum effects finds its proper expression in the circumstance that any attempt of subdividing the phenomena will demand a change in the experimental arrangement introducing new possibilities of interaction between objects and measuring instruments which, in principle, cannot be controlled” [20] (v. 2, p. 39). Any such subdivision only creates new phenomena, rather than allows one to reach quantum objects. In his reply to Schrödinger, Bohr says:

However, these considerations [of the atomic structure of instruments] do not have any close connection to the Einstein [EPR] paradoxes and to the question of limitation of the [classically] causal description of quantum phenomena. On this point I must confess that I cannot share your doubts. My emphasis of the point that the classical description of experiments is unavoidable amounts merely to *the seemingly obvious fact that the description of any measuring arrangement must, in an essential manner, involve the arrangement of the instruments in space and their functioning in time, if we shall be able to state anything at all about phenomena*. The argument here is of course first and foremost that in order to serve as measuring instruments, they cannot be included in the realm of application proper to quantum mechanics. [25] (v. 7, p. 511, Letter to Schrödinger, 25 October 1935)

In other words, measuring instruments in their observable parts are available even to our immediate phenomenal perception. Indeed, they must be available to it or at least to thought for us to “*be able to state anything at all about phenomena*” and, thus, to unambiguously communicate our findings, along with the mathematics that predicts them (equally available to thought), to meet “basic requirements of science” [12] (p. 697). This also makes CP essential in QP, including for a functional QT, such as QM, to predict anything, while, in RWR interpretations, precluding any physical connections other than predictions (which is “the realm of applications proper to quantum mechanics”) between QM and what is observed. On the other hand, the ultimate nature of the reality responsible for observed phenomena may be and, in Bohr’s RWR interpretation, is beyond the reach of thought and, hence, nothing about it can be communicated. The same situation is found in high-energy (QFT) regimes, whether we will ever be able to include the atomic constitution of measuring instruments in the theory or not. As Heisenberg says, following Bohr’s argument, and aware of Bohr’s exchanges with Einstein and Schrödinger on the subject:

[It] has sometimes been suggested that one should depart from the classical concepts altogether and that a radical change in the concepts used for describing the experiments might possibly lead back to a nonstat[ist]ical [sic!], completely objective description of nature…. This suggestion, however, rests upon a misunderstanding. The concepts of classical physics are just a refinement of the concepts of daily life and are an essential part of the language which forms the basis of all natural science. Our actual situation in science is such that we do use the classical concepts for the description of the experiments, and it was the problem of quantum theory to find theoretical interpretations of the experiments on this basis. There is no use in discussing what could be done if we were other beings than we are. At this point we have to realize, as von Weizsäcker has put it, that “Nature is earlier than man, but man is earlier than natural science”. The first part of the sentence justifies classical physics, with its ideal of complete objectivity. The second part tells us why we cannot escape the paradox of quantum theory, namely, the necessity of using classical concepts. [43] (p. 56)

In fact, there is no paradox. Classical concepts reflect the essential workings of our biological and specifically neurological constitution developed in our evolutionary emergence as human animals. Our general phenomenal thinking, as the product of this constitution, is classical in the sense that it is consistent with and leads to the concepts of CP. Any concept we form is derived from and can only apply to observed phenomena, and as observed phenomena, quantum phenomena are physically classical. They are different from classical phenomena only because the data observed in them precludes us from describing how they come about or predicting these data by CP. In RWR interpretations, however, while QT (QM or QFT) fully adequately predicts these data, how these data come about is not described by QT or even conceivable. Conceiving of the emergence of these data may, in principle, be precluded by the same evolutionary nature of ours and, thus, by our thinking and language, developed in the interaction with (classical) objects consisting of millions of atoms, rather than anything on the atomic scale (e.g., [44] (p. 11)). This is a manifestation of the fact that human thinking is “earlier than natural science” and limits the latter. As discussed below and as Heisenberg was aware, it is not a matter of size. Quantum objects could be macroscopic, as is, for example, a Bose–Einstein condensate or a Josephson device. It is a matter of their quantum behavior defined by their microscopic constitution; a behavior manifested in their effects on quantum observational instruments. Only a special instrument can detect either object as quantum, as having quantum effects, which QT can predict. QP takes us much further away from our general phenomenal thinking and, unlike CP, is even incompatible with it if one also excludes abstract mathematics from our general phenomenal thinking [6] (pp. 83–86). QP is a product of a specific technology, beyond our bodies, built by us (or something in nature that we use as our instruments), while QT is a form of mathematics created by our thoughts and, neurologically, our bodies. Our thought is also necessary for creating any technology or using something in nature as technology.

These considerations do not imply that new concepts are not possible in QT. QM and QFT would not have been possible without the invention of new concepts, any more than earlier physical theories, beginning with classical or even Aristotle’s physics, would have been. This invention defines the creative practice of all physics, just as it does mathematics or philosophy, keeping in mind that new physical variables or (which is often forgotten) new equations are also new concepts. The very creation of QM and QFT was driven by mathematical concepts, such as Heisenberg’s matrix variables (in essence, Hilbert space operators), expressly introduced without being representations of classical concepts. This, I argue, is characteristic of QT, which, by the same token, can only be established as different from CT mathematically. (Quantum phenomena can be established experimentally apart from any theory). It is true that the wave function was initially introduced by Schrödinger with such a representation in mind. Apart, however, from the major difficulties of using the idea of quantum waves as something physical, the wave function was quickly given a probabilistic interpretation by Born, which only related to it as a mathematical feature of formalism. In fact, this interpretation makes the term “wave function” obsolete, vs. a more common and more suitable ψ. Schrödinger’s entanglement was also a mathematical concept of the formalism of QM. It cannot be represented by a physical concept, at least in RWR interpretations, which place the ultimate reality responsible for what is predicted by means of entanglement beyond representation or even conception. Bohr’s concepts of complementarity and phenomenon are, by contrast, new physical concepts, representing, however, quantum phenomena, independently of QT, and these concepts only rely on the mathematics of CP. The question is whether one can avoid classical physical concepts or CP or whether new realist concepts, describing the ultimate nature of the reality responsible for quantum phenomena are possible or even necessary, as both Einstein and Schrödinger thought. On the first question, Bohr’s view—or the present view—is that classical concepts and CP cannot be avoided. On the second question, as noted, Bohr’s answer, or at least that of the present author, would be that new realist concepts or theories representing the ultimate reality responsible for quantum phenomena, may not be possible, which is not the same as that they are not possible.

Schrödinger was, however, not yet finished in his letter, and asked another question, which surprised Bohr as revealing something in Bohr’s thinking of which Bohr was not entirely aware himself at the time, and which is perhaps the most perceptive part of Schrödinger’s letter:

However that may be [concerning a possible removal of the classical description of observation in relativistic quantum regimes], there must be clear and definite reasons which cause you repeatedly to declare that we *must* interpret observations in classical terms, according to their very nature. Whenever you say that, you state it so definitely and clearly, in the indicative, without any reservation like “probably”, or “it might be”, or “we must be prepared”, as if this were the uttermost certainty in the world. It must be among your firmest convictions—and I cannot understand what it is based upon.

It could not be just the point (about which you talked so insistently to me already in 1926): that our traditional language and inherited concepts were completely unsuited to describe the phenomena with which we are concerned now. Because, in the course of the development of our science (and mathematics), from its earliest beginning to the situation at the end of the nineteenth century this was certainly the case over and over again. If the break with the old tradition seems greater now than ever before, then we should take into account that a particular time perspective is responsible for forming the impression *that* developments in which we ourselves take part, stands out as being more important and more essential that earlier ones, which we cite only from history, and whose stages we get to know mostly in reverse order. In fact, it is often difficult for us to imagine *earlier* ways of thinking. And although the difficulty of such a historical step *back* actually speaks most eloquently of *how* significant [the step] must have seemed to the pioneers of their earlier advances, still now and then we cannot avert feeling: “Incredible that, up to then, people were so narrow-minded!” Here, the underestimation of the time perspective shows itself most clearly.

Thus I think that the fact that we have not adapted our thinking and our means of expression to the new theory cannot possibly be the reason for the conviction that experiments must always be described in the classical manner, thus neglecting the essential characteristics of the new theory. [25] (v. 7, pp. 508–509, Letter to Bohr, 13 October 1935)

Indeed, as Bohr’s reply to Schrödinger suggests, this is not the reason. It is not a matter of going beyond a tradition, say that of CT or even pre-quantum-mechanical QT. It is difficult to object, and Bohr would not, to what Schrödinger says on this point. It would, however, also be difficult to agree that Bohr had ever neglected the essential characteristics of QM; quite the contrary, he affirmed them, not the least, as essentially different from CT, either deterministic or probabilistic. Bohr’s emphasis on the classical description of the observed parts of measuring instruments is one of the essential characteristics of QT in his interpretation or that of the present author. It is not that “we have not adapted our thinking and our means of expression to the new theory”. In fact, physicists had adapted their thinking (in terms of physical and mathematical concepts) to QT, and Bohr was one of the first to do so. The reason for the conviction that “the experiments must also be described in a classical manner” was stated by Bohr in his reply: “*the seemingly obvious fact that the description of any measuring arrangement must, in an essential manner, involve the arrangement of the instruments in space and their functioning in time, if we shall be able to state anything at all about phenomena*”. What is observed in experiments even must be so described because: “the argument here is of course first and foremost that in order to serve as measuring instruments, they cannot be included in the realm of application proper to quantum mechanics”. This is far from “neglecting the essential characteristics on the new theory”, QM.

Bohr’s “*must*” in “we *must* interpret observations in classical terms” is stated “so definitively” and “without any reservation” for the following reason. While QM may become obsolete one day (although it has remained in place for a century now, so not anytime soon in this author’s view), so too might RWR interpretations of it in favor of realism; however, the necessity of interpreting observations in classical terms will remain. Schrödinger was astute to notice Bohr’s “must”, as Bohr did not fail to acknowledge in his reply: “I found it most amusing that you noticed—which I myself had not at all been aware of—that just on this point, and only on this one, I do not say, ‘it might be’” [25] (v. 7, p. 512, Letter to Bohr, 13 October 1935). Bohr will become more aware of this fact from this point on, with its significance even more pronounced in his subsequent writings. Bohr’s “must be” reflects his assumption of the necessity of unambiguous, and in this sense objective, communication of the outcomes of experiments, insured by the classical description of the observable part of measuring instruments. As Bohr said later (in 1949):

It is decisive to recognize that, however far the phenomena transcend the scope of classical physical explanations, the account of all evidence must be expressed in classical terms. The argument is simply that by the word “experiment” we refer to a situation where we can tell others what we have done and we have learned and that, therefore, the account of the experimental arrangement and the results of the observation must be expressed in unambiguous language with suitable application of the terminology of classical physics. [20] (v. 2, p. 39)

The same unambiguous communication is possible and necessary in the case of the logical and mathematical structures of QM or QFT. Bohr brings these two forms of unambiguous communication together in another passage, reflecting his persistent concerns with this problem:

When speaking of a conceptual framework, we refer merely to the unambiguous logical representation of relations between experiences. … A special role is played by mathematics which has contributed so decisively to the development of logical thinking, and which by its well-defined abstractions offers invaluable help in expressing harmonious relationships. Still, … we shall not consider pure mathematics as a separate branch of knowledge, but rather as refinement of general language, supplementing it with appropriate tools to represented relations for which ordinary verbal expression is imprecise and cumbersome. In this connection, it may be stressed that, just by avoiding the reference to the conscious subject that infiltrates daily language, the use of mathematical symbols secures the unambiguity of definition required for objective description.

The development of the so-called exact sciences, characterized by establishing of numerical relationships between measurements, has indeed been decisively furthered by abstract mathematical methods originating from detached [from more personal or “subjective” concerns] pursuit of generalizing logical constructions. This situation is especially illustrated in physics. [20] (v. 2, p. 68)

As I argue here, the mathematical–experimental character of modern physics is even more defined by mathematical constructions of physical theories enabling such numerical relations, thus making the mathematical formalism of these theories define their experimental nature, in accord with Heidegger’s view [9] (p. 93). QT (or relativity) requires both CT and QT (or CT and relativity) and, thus, two different, including mathematical, forms of physical theories. In any type of CT, only one theory is required.

As indicated earlier, it is sometimes argued that, while possibly necessary for the description of the observable quantum phenomena and measurements associated with them, CP is not a separate theory but a limited case of QM and, hence, not a fundamental theory. Most of these arguments contend that the observable parts of measuring instruments can be handled by QM, without, in contrast to Schrödinger’s view, contending that the situation requires new concepts, beyond those of QM (although Schrödinger, too, sees the cat as a quantum object in the cat experiment). These arguments, at least those I am familiar with, do not appear to me to be convincing, but being unable to properly consider them here, I shall refrain from definitive assessment of them. In any event, this is not the present view or that of Bohr, which only allows that in certain circumstances quantum objects can be considered by means of CT, which is a very different claim. In the present view, the cat in the cat experiment is always a classical object that cannot, by itself, be handled by QM. QM is only applicable for predicting the outcome of the cat experiment because the overall arrangement involves properly quantum objects and events: the emission of a particle by a radioactive atom and the interaction between this particle and the Geiger counter used.

Our probability assignments concerning outcomes of quantum experiments may be different, depending on different information we have pertaining to a given experiment, and in this sense, they are subjective or personal. The latter might be a better concept as these assignments are also shaped by things in the world, such as observational instruments or the world itself, which is assumed in this article or by Bohr to exist independently and, thus, to be external to an agent, or other factors. Things are rarely, if ever, completely subjective, permitting that such exterior factors are interiorized at the time of an assignment of one or another probability to a future event. In any event, a quantum experiment that would be performed would, by virtue of its classically describable outcome, give a definitive and unambiguously communicable result, and as such, objective, information. As Bohr said in the statement cited above, “By the word ‘experiment’ we refer to a situation where we can tell others what we have done and we have learned” and where we can do so unambiguously [20] (v. 2, p. 39). By contrast, Bohr persistently refers, as in his counterarguments to EPR, to “the essential ambiguity involved in a reference to physical attributes of objects when dealing [as in QP] with phenomena where no sharp distinction can be made between the behavior of the objects themselves and their interaction with the measuring instruments” [20] (v. 2, p. 61). An agent cannot control the outcome of a quantum experiment but can only predict it probabilistically by means of QM (cum Born’s rule). One might be able to decide which observation or measurement to perform, for example, one or the other of two complementary observations, thus also defining which predictions to make. One cannot, however, in general, control the outcome of this experiment concerning the value one obtains when performing the measurement defining this outcome. In addition, one can always perform an alternative, complementary, measurement at the endpoint of the experiment, which will irrevocably disable the original estimate.

This makes measurement objective in this double sense—the lack of control of a future outcome and the possibility of an unambiguous communication of any actual outcome—without, in the present view, objectively attributing anything to nature itself, except from its existence and, as part of it, human existence. Making an observation or measurement is, in the present view, a unique act of creation with a unique outcome, an act that can be performed by a particular agent or several agents. As such it has subjective or, again, personal aspects, including those shaping our decision concerning this action. Once the measurement is performed, however, the outcome becomes a permanent record, part of the archive of physical data, always classical and as such available to thought or even our immediate phenomenal perception. It may be unknown to others, but it is not the same as being subjective. It is also true that, as with any record, it must still be experienced by us or others to be meaningful. In other words, performing an act of observation or measurement is personal (if sometimes determined collectively), but its outcome need not be. It can also be experienced or interpreted differently by different agents.

Science is a human enterprise. However, sharing and communicating our estimates (particularly numerical parts of them) of possible events and experiences is also human, and doing so is helpful and even unavoidable in human life. Science, as Bohr noted in the passage cited above, capitalizes on this fact and on the possibility that the communication involved may be made unambiguous, helped by the use of mathematical symbols. These symbols or their organizations, including those of the mathematical formalism of QM or QFT, are also unambiguously communicable. Mathematics, as a discipline, depends on this fact. This possibility is not restricted to mathematics or exact mathematical sciences, but is, as E. Husserl argued, inherent in human thinking, language, and culture [45] (p. 66). Mathematics and exact sciences capitalize on this possibility, while still retaining personal, including personalized, even concerning scientific concepts, aspects of experience, especially when it comes to probability. CP is more open to this personalization, as it is more closely connected to our general phenomenal experience.

According to Bohr, “when speaking of a conceptual framework, we refer merely to the unambiguous logical representation of relations between experiences” [20] (v. 2, p. 68). It is not coincidental that Bohr speaks of experiences, which may be mathematical, rather than only of experiments. These experiences always combine individual and shared or communicable aspects (arguably better terms than “subjective” and “objective”), with mathematics and science aiming to maximally reduce the ambiguity of defining and communicating what needs to be so shared. In CP and relativity, how the outcomes of experiments come about is visible to thought as well, at least in the case of relativity, mathematical thought. What is observed there may also be assumed to exist independently of observation, for all practical purposes, but, in the present view, only for all practical purposes, defined by human agents and agencies, such as science [2] (pp. vii–xxiv). This independence is no longer possible in QP, where the difference between quantum phenomena and the ultimate reality responsible for them, including quantum objects, is irreducible, regardless of interpretation. In QT in RWR interpretations, however, the recourse to probability, correlative to the irreducible dependence of quantum phenomena on the agencies of observation, is no longer due, as in CP, to our insufficient knowledge of how the phenomena considered come about. It is due to the impossibility of any conception of how these phenomena come about.

I do not know what Schrödinger thought upon receiving Bohr’s reply, although it does not appear that he ever accepted Bohr’s view concerning the irreducible role of classical concepts in QP. It is not clear, for example, to what degree, if any, the cat paradox or Schrödinger’s paper overall were attempts to show that new physical concepts may be necessary in QT. As noted, he assumed that the cat was a quantum object, as suggested by his statement that “the *ψ*-function of the entire system would express this by having in it the living and the dead cat (pardon the expression) mixed or smeared out in equal parts” [1] (p. 157). This view would not be Bohr’s view for the reasons that must be apparent from the preceding discussion and is further explained in the next section. In any event, Schrödinger, as did Einstein, thought that the argument that new concepts associated with quantum objects and their behavior received new support from the EPR experiment. Such concepts, they thought, would ground a realist alternative to QM, which, as noted, was by then viewed by Schrödinger as “perhaps after all a convenient calculational trick” [1] (p. 167).

Neither Einstein nor Schrödinger thought that QM was likely to be interpreted on realist lines, although such interpretations have been advanced. Both thought that the EPR experiment showed that QM is, if local (no action at a distance), ultimately incomplete as a description of individual quantum processes or events, because it could not predict anything that could be established as real [1,11] (pp. 163–166). Bohr argued that EPR’s argument was unsustainable, thus allowing one to maintain that QM is both local and complete, at least as things stood then, but little has changed in this regard since) [2,12] (pp. 227–257). Bohr did not appear to have considered Schrödinger’s analysis of the EPR experiment in the cat paradox paper. This analysis, however, was essentially a reprisal of EPR’s argument and, thus, would have allowed for Bohr’s counterargument. The debate concerning the EPR and related experiments and the Bohr-EPR exchange is still ongoing. Whether one accepts or not Bohr’s counterargument, for Bohr, the EPR experiment confirmed “how far, in quantum theory, we are beyond the reach of pictorial visualization”, far enough to be beyond the reach of thought, making the reality ultimately responsible for quantum phenomena invisible to thought [20] (v. 2, p. 59).

## 4. Visible and Invisible, and Classical and Quantum in Schrödinger’s Cat Experiment

Schrödinger’s paper containing the cat experiment offered an overall investigation of QM, as reflected in its title “The present situation in quantum mechanics” [1]. It was also a response to EPR’s paper, which Schrödinger discusses at some length, and was arguably most important for the concept of entanglement, prompted by the EPR experiment. The paper had two companion papers [16,46]. Schrödinger’s analysis of QM is penetrating, even though (or because) Schrödinger assessed QM, especially as interpreted along RWR lines, negatively as “a doctrine born of distress” and “perhaps after all only a convenient calculational trick” [1] (pp. 154, 167). Schrödinger had worries about the theory as primarily suited for calculations rather than a true account of the workings of nature as early as 1926 in introducing the time-dependent Schrödinger’s equation. The latter used, for the first time, the complex-valued wave function. Schrödinger was unhappy about this because it complicated a representational role that he wanted to give to wave mechanics [47] (p. 123) [2] (pp. 157–159). His hopes for doing so did not materialize during the intervening decade. As indicated above, EPR’s argument appeared to Schrödinger, as it did to Einstein, to show that QM, while correct, was incomplete or else nonlocal, justifying their view that an alternative, hopefully realist, theory of quantum phenomena was necessary. Neither accepted nor, it appears, followed Bohr’s counterargument. Indeed, Einstein appears to have misread it by thinking that Bohr assumed that QM was nonlocal, while Bohr argued that it was both complete and local [2] (pp. 248–259).

The cat experiment was part of Schrödinger’s overall analysis of QM, albeit a relatively marginal part that did not initially receive much attention, neither did the paper itself (initially), nor the concept of entanglement. During the last half a century or so, however, the cat experiment has been endlessly discussed, with ever-proliferating interpretations (often along with those of QM itself) in technical, philosophical, and popular literature, and has acquired a semi-mythical status, as manifested, for example, in [48] and innumerable other commentaries. There are many reasons for this attention, such as its role in helping realist views of QM or supporting hopes for realist alternatives, or in countering the Bohr postulate, often resisted as much as RWR interpretations (e.g., [4]). (An instructive realist analysis of the cat experiment, as well as the Wigner friend experiment, is given in [49] (pp. 89–94)). There is also a narrative appeal of the experiment, overly exploited in popular accounts.

From the present perspective, there is nothing especially revealing in the cat experiment, or that would challenge RWR interpretations, such as Bohr’s or the present one, or the Bohr postulate. In fact, as I argue here, it may be seen as supporting the Bohr postulate, which is my main reason for considering the cat experiment here. As I said, there does not appear to be any evidence that Bohr ever commented on the experiment or Schrödinger’s paper. The letter exchange, discussed above, between Schrödinger and Bohr concerning Bohr’s emphasis on the role of CP in QM is relevant to the cat experiment. But this exchange took place before Schrödinger’s paper was finished, and as the preceding discussion makes clear, it was about Bohr’s views. I surmise that Bohr would not find anything challenging his views in the cat experiment. I also surmise that he would have been likely to see the cat as a classical and not a quantum object. The Dirac postulate lends support to this view. By the Dirac postulate, each quantum observation registers a different quantum object, merely allowing one to assume that successive observations deal with the same quantum object as a statistically permissible idealization in low-energy quantum regimes. By contrast, the cat is always the same object, in a different classical state, at any stage of the experiment. Schrödinger describes the experiment as follows:

One can even set up quite ridiculous cases. A cat is penned up in a steel chamber, along with the following diabolical device (which must be secured against direct interference by the cat): in a Geiger counter there is a tiny bit of radioactive substance, so small, that perhaps in the course of one hour one of the atoms decays, but also, with equal probability, perhaps none; if it happens, the counter tube discharges and through a relay releases a hammer which shatters a small flask of hydrocyanic acid. If one has left this entire system to itself for an hour, one would say that the cat still lives if meanwhile no atom has decayed. The first atomic decay would have poisoned it. The ψ-function of the entire system would express this by having in it the living and the dead cat (pardon the expression) mixed or smeared out in equal parts. [1] (p. 157)

In the present interpretation, the last sentence would not apply, and it appears to be contradicted by the preceding two sentences, which are in accord with the present interpretation. First, however, Schrödinger adds an elaboration that is rarely given proper attention but that provides a further context for the cat experiment. He says: “It is typical of these cases that an indeterminacy originally restricted to the atomic domain becomes transformed into macroscopic indeterminacy, which can then be resolved by direct observation. That prevents us from so naively accepting as valid a ‘blurred model’ for representing reality” [1] (p. 157). A blurred model is defined by a view of the ψ-function as “an imagined entity that images the blurring of all variables at every moment [unless a measurement intervenes] just as clearly and faithfully as the classical model its sharp numerical values” [1] (p. 156). In other words, the ψ-function should not be seen as representing the independent behavior of quantum systems as blurred. So, it is possible that Schrödinger does not subscribe to the view that the cat is in a superposition and sees the experiment as countering the blurred model. The case is, however, not clear because there is another reference to the cat experiment [1] (p. 167) that suggests that he does assume that “the living and the dead cat [is] mixed or smeared out in equal parts” before the box is opened. This is a common reading of Schrödinger’s view and a common assumption in interpreting the experiment in general.

As must be expected from the preceding discussion, the present view of the cat experiment is entirely different. First, in the RWR interpretation, defining this view, the ψ-function does not represent even the behavior of properly quantum objects (or the ultimate reality responsible for quantum phenomena) either. It only provides or more accurately (because one needs Born’s rule) enables, in Schrödinger’s phrase, an “expectation-catalog” for possible future experiments [1] (p. 154). Secondly, as I argue here, there is no good reason to assume that the cat is a quantum object. One can only use QM to make predictions concerning its state as being alive or dead at a certain point in the experiment, because its (always) classical state is linked to properly quantum objects within the overall arrangement. If considered by itself, the cat would always be either dead or alive at any stage of the experiment, even without us knowing which before opening the box. This is, in fact, implied by Schrödinger’s sentences preceding the one claiming that the cat is in a superposition of being dead and alive: “If one has left this entire system to itself for an hour, one would say that the cat still lives if meanwhile no atom has decayed. The first atomic decay would have poisoned it”. Why then claim that “the ψ-function of the entire system would express this by having in it the living and the dead cat (pardon the expression) mixed or smeared out in equal parts”?

It is, in principle, possible that by saying that “the ψ-function of the entire system *would express this by having in it*” (emphasis added) Schrödinger only meant the mixing of amplitudes for these two outcomes. Then, “the ψ-function of the entire system” would merely allow for both possible future outcomes, in accord with the present view. Schrödinger, however, does not qualify his statement in this way. In the present view, without any conflict with the first two sentences, which would refer to a classical object, what will be so “mixed” are mathematical state-vectors in the formalism. This mixture enables the probability of predicting the atomic decay involved, to which such terms as “dead” or “alive”, or any other terms, including “to be”, do not apply. It is only by using this purely mathematical mixture that one can estimate the probability of finding the cat dead or alive, depending on the proper quantum event of an atomic decay. The ψ-function has no association with the cat apart from these predictions and does not represent the state of the cat as a classical object. In RWR interpretations, the ψ-function would not represent the physical state of a purely quantum object either, whether assumed to exist independently or defined, still as beyond conception, at the time of observation by the Dirac postulate.

There is nothing that can be said or thought about the *ultimate* reality responsible for quantum phenomena, such as that responsible for the atomic decay in the cat experiment. By contrast, there are always things we can say about any classical reality involved in quantum experiments, as part of what is, *in principle, observable* in them, as is the cat in the cat experiment. My “in principle observable” deliberately echoes Heisenberg’s famous and much misunderstood opening claim in his first paper of QM that his new mechanics is grounded in “the *relationships* between quantities which in principle are observable” [50] (p. 263). These quantities are empirically observable in the instruments used, but these *relationships* (the word often disregarded in empiricist readings of this statement) are the probabilistic relationships established by his new mechanics. As Heisenberg said, shortly before completing his paper: “What I really like in this scheme is that one can really reduce *all interactions* between atoms and the external world … to transition probabilities” (Letter to R. Kronig, 5 June 1925; cited in [51] (v. 2, p. 242)). Speaking of the “interactions between atoms and the external world” implied that QM was only predicting the effects of these interactions observed in the instruments used, without representing how these effects come about. As explained here, this procedure replaced measurement in the classical sense (of measuring preexisting properties of objects) with establishing quantum phenomena, which can be treated classically, without measuring the properties of quantum objects.

One could, again, in the arrangement of the cat experiment, consider the cat as *part* of an object under investigation, concerning which the prediction in question can be made by means of QM. As noted earlier, however, the cat can only be part of this object because a proper quantum object must be involved to have a quantum experiment and to use QM. By a (properly) quantum object, to reiterate, I mean an object that can be treated as quantum when considered as such, by itself, independently of any other object, as an electron or a photon could be. In the cat experiment the key properly quantum object is a “particle” emitted by radioactive decay, which, as the atom that decays, can always be treated as a quantum object by itself. The cat, made of atoms as it is, cannot be treated as a quantum object if considered by itself. (The radioactive atom itself is also a quantum object, but its role in the arrangement, while necessary is secondary, as one could stage the experiment by using a cosmic ray particle). The cat experiment is a quantum experiment because of the particle created by radioactive decay and not because of the cat. Therefore, considering the cat as part of an object under investigation by means of QM does not change the point that the cat is a classical object, always available to thought or even to our immediate sense perception, before and after the experiment. A (properly) quantum object is never available to our sense perception and, in strong RWR interpretations, to our thought. One can at any point see the cat as such, independently of a quantum observational device, by opening the box or using the box with glass walls. One can never, physically (and, thus, regardless of interpretation) detect by our sense perception a properly quantum object or rather establish its presence without a suitable observational device. A quantum object, or the ultimate reality responsible for quantum phenomena (of which this object is part), cannot be observed as separated from the phenomenon considered, which is a result of the interaction between this object and the instrument.

It is, accordingly, more reasonable to see the cat as a classical object, which is part of the combined object under investigation in the cat experiment. Because of the presence of properly quantum components (the radioactive atom and the particle it emits), QM enables one to predict the cat’s possible classical state of being dead or alive at the final stage of the experiment. It may be added that the state of the cat has nothing to do with, in Bohr’s language, the “irreversible amplification” of the interaction between an instrument and a quantum object to the classical level of observable effects [20] (v. 2, p. 73). These effects, as noted, are also seen in terms of “decoherence”. Either view would only pertain to the interaction between the emitted particle and the Geiger counter and the resulting classical effect, which leads to the release of the poison. The cat is a classical object, and its state, dead or alive, has nothing to do with either view at any stage of the experiment.

As such the cat can also be described by ordinary language, as opposed to a properly quantum object, with which no specific concept can be associated, in RWR interpretations. As Heisenberg said:

There is no description of what *happens* to the system between the initial observation and the next measurement…. The demand to “describe what happens” in the quantum-theoretical process between two successive observations is a contradiction in adjecto, since the word “describe” refers to the use of classical concepts, while these concepts cannot be applied in the space between the observations; they can only be applied at the points of observation…. [T]he problems of language are really serious. We wish to speak in some way about the structure of the atoms [as made of particles] and not only about ‘facts’—the latter being, for instance, the black spots on a photographic plate or the water droplets in a cloud chamber. However, we cannot speak about the atoms in ordinary language. [43] (pp. 57, 145, 178–179)

Nor is it possible in terms of ordinary concepts, from which ordinary language is indissociable. This fact inhibits and, especially in RWR interpretations, ultimately precludes the use of specifiable concepts, such as, “spinning” or rotating, in the case of spin vs. numerical values, the direction of the pointer used (commonly up or down) as observed in our instruments, or metaphors, which relate concepts, possibly from different domains. Technical, philosophical, and popular thinking or expressions rely on metaphors to convey what “happens” at quantum micro scales, such as “a cloud of virtual gluons” inside a nucleus. Indeed, the “nucleus” of an atom is already a metaphor. In considering, however, what happens between quantum experiments, both ordinary (or even physical) concepts and metaphors can only serve as heuristic guides and help explanations. Sometimes ordinary language or concepts and metaphors even enable explanations, especially in nontechnical accounts, which cannot rely on the mathematics of QM or QFT. But at least in RWR interpretations of QM or QFT, mathematics, too, only serves for predicting numbers associated with what is observed in our instruments and does not explain how the phenomena with which we can associate these numbers come about. On the other hand, as Bohr argues, the use of ordinary language and concepts is not only possible but also necessary in representing the “facts” to which Heisenberg refers, and in communicating them. As discussed earlier, in physics or other sciences one can and must do so unambiguously, as unambiguously as possible. The same is true in mathematics, where bypassing ordinary language and concepts is possible to a greater degree, even if not completely.

Unlike Bohr, Heisenberg at the time of the above statement allowed that the ultimate nature of the reality responsible for quantum phenomena could be represented mathematically [43] (pp. 59–75, 145, 167–186) [2] pp. 75–76. The words “happens” or “physical”, or any words, even to “exist” or to “be”, need no longer be part of this representation, which would only require mathematical symbols, possibly defined with the help of ordinary language. It is hard to avoid “to be”. A (more) purely mathematical representation is also possible in CP, for example, by means of Hamiltonian or Lagrangian formalism. In CP, however, it remains possible to speak about such behavior in terms of physical or even ordinary concepts, such as motions, trajectories, and so forth. This is no longer so in RWR interpretations of QT. In QT, especially in QM and QFT, mathematics replaces language, including metaphors, to the greatest degree ever, making the theory more akin to abstract mathematics itself. This, in this respect also liberating, role of mathematics, has always been one of Heisenberg’s key points [44] (p. 11). His argument concerning the mathematical, vs. physical or linguistic, representation of how quantum phenomena come about is, however, fully consistent with Bohr’s view that common language and classical physical concepts remain essential in dealing with experiments. This is quite clear from his comments, cited above, about phenomena factualy observed in quantum experiments. To reprise Bohr’s statement in his exchange with Schrödinger: “My emphasis of the point that the classical description of experiments is unavoidable amounts merely to *the seemingly obvious fact that the description of any measuring arrangement must, in an essential manner, involve the arrangement of the instruments in space and their functioning in time, if we shall be able to state anything at all about phenomena*”. He adds, however, “the argument here is of course first and foremost that in order to serve as measuring instruments, they cannot be included in the realm of application proper to quantum mechanics”. In other words, CP is necessary to describe quantum phenomena but is never sufficient for accounting for them, most crucially as unable to predict them, which requires a QT, such as QM or QFT.

Such concepts as “dead” or “alive”, describing possible states of the cat, cannot apply to quantum objects, unless metaphorically, as when speaking about the birth and disappearance of “particles” (ultimately a metaphor as well in dealing with quantum objects) in QFT. One can indeed say that if one can actually, rather than provisionally or metaphorically, describe an object by means of language and concepts, this object is classical, by which, as noted, I mean an object, in principle, allowing for being idealized by CT. A state of a classical object can, again, be predicted by QM under certain circumstances, in which, however, it must be part of a combined system containing a properly quantum object, such as an emitted particle in the cat experiment. There is no problem of ever speaking about the cat at any stage of the experiment, even though we do not know whether it is dead or alive before we open the box. We always know that it is either one or the other even then, as would be confirmed if we use the box with glass walls. Hence, as I argue, Schrödinger’s claim that “the ψ-function of the entire system would express this by having in it the living and the dead cat (pardon the expression) mixed or smeared out in equal parts” is problematic and is even inconsistent with what he says just before this statement: “If one has left this entire system to itself for an hour, one would say that the cat still lives if meanwhile no atom has decayed. The first atomic decay would have poisoned it” [1] (p. 157). This statement clearly implies that the cat is in a definite state, dead or alive, at any point of the experiment. The statement “the ψ-function of the entire system”, where “the entire system” is an important qualification, conveys something else. It conveys a specific quantum-mechanical way of estimating the probability of finding it either dead or alive after a certain interval of time when we open the box, or when we look inside the box with glass walls. Using glass walls makes no difference in assessing these probabilities, defined by other, properly quantum parts, of “the entire system” (the radioactive atom and the emitted particle).

It is possible to stage a purely quantum version of the cat experiment by replacing a cat with a properly quantum object in an arrangement in which a radioactive decay or the absence thereof would lead to two alternative “Schrödinger” (physical) states, *a* or *b*, of this object, as detected, unavoidably classically, by a quantum observational instrument. This is, however, a completely different experiment. First, in this case, as quantum, the object, even if macroscopic, cannot, in principle, be observed, in either state, *a* or *b*, as such by itself, in the way the cat could be. As properly quantum, such an object, I argue, requires an instrument to be detected by an observation with a classical outcome. Hence, using a box with glass walls, which would allow one to see the cat at any point, will make no difference in this case, because one cannot see this quantum object as such. If this object is macroscopic, as a Josephson device, it can only be seen as a classical object (two superconductors standing in some lab). This difference confirms that one cannot say, as some do, that using the box with glass walls in the cat experiment changes the experiment. It is logically clear that, even if one cannot physically see the cat inside of the box, one can still “see” the cat in one’s mind eye as a classical object, either dead or alive, even if one does not know which. One could also confirm it by opening the box at any point, although, admittedly, this still allows one to assume that the cat is a quantum object before one does so. It would be intriguing to consider this type of experiment with C_60_ molecules that can be observed as both classical and quantum objects, which would exemplify two situations here considered, but would not change the argument offered here. One would never be able to observe them as quantum without a quantum instrument, or, since, as quantum, they cannot be observed, detect them through their quantum effects.

The claim that the cat is a classical object is falsifiable (even if, in my view, unlikely to ever be falsified), by installing some quantum detectors that would detect it as a macroscopic quantum object, an instrument always necessary to do so, as in the case of a Josephson junction or a Bose–Einstein condensate. As noted, distinguishing classical and quantum macroscopic, or microscopic, entities or strata of reality is not a matter of their size but of their constitution manifested in different types of effects, beginning with the role of *h*. There are, however, other specifically quantum effects. It is sometimes argued that the reasons that we cannot observe classical objects, such as a cat, as quantum, are merely practical (in the case of objects like cats possibly practically insurmountable), rather than fundamental. It has been contended, for example, via the concept of decoherence, that this has to do with the difficulties of isolating them from their environments, which prevents one from performing suitable quantum experiments to “see” classical objects as quantum (e.g., [52] (pp. 4–5) and further references there). There is, however, no real evidence, thus far, supporting this argument; and as noted, while having its strong (and vocal) advocates, the decoherence theory is not commonly accepted. Some physical objects might be classical for fundamental reasons. There may be no situation and no instrument that will make it, in principle, possible to detect them as (properly) quantum objects.

Given that the ultimate constitution of nature is generally assumed to be defined by elementary particles, or fields, as quantum objects, the classical nature of some physical objects may appear enigmatic. From the present perspective, however, this classical nature has to do with the nature of our interactions with the world by means of our bodies, specifically our brains, and thinking. These interactions make us phenomenally “see” the world as classical, as expressed here by the Bohr postulate. At the same time, in strong RWR interpretations, we cannot represent or even conceive of the ultimate constitution of the reality responsible for quantum phenomena. We need, and fortunately for physics, have been able to create, the technology to detect these effects and the mathematics to predict them, even if only probabilistically, which is, again, fully in accord with what is experimentally possible thus far. By the same token, we cannot “see” the ultimate, “quantum”, constitution of classical objects either, that is, objects defined as classical in our interaction with nature, rather than existing as such in nature itself. In strong RWR interpretations, the ultimate constitution of nature is beyond conception in view of the existence of quantum phenomena, defined by our interactions with nature. These interpretations preclude us from saying how nature ultimately is. They are, however, fully in accord with the fact that nature allows us to have physics by connecting, in Bohr’s phrase, “the manifold aspects of our experience”, in a specific mathematical–experimental way, which may be classical, relativistic, or quantum [20] (v. 1, p. 18).

Bohr’s invocation of experience, as noted earlier, is crucial because it is ultimately our experience that makes science or mathematics possible, but we are also able to establish science or mathematics through a specific form of thinking and practice. Of course, asking how nature ultimately is might be part of this thinking and practice, just as assuming that it is not possible to know—or even conceive—how nature ultimately is, as in strong RWR interpretations. By the same token, such interpretations, too, could only be supported and indeed defined by our interactions with nature by means of technology and mathematics, and not by nature itself beyond us. We are part of nature, but a special biological part, distinguished in addition by our specific capacities of interacting with nature, possibly only shared by other animals biologically related to us by evolution. RWR interpretations allow us to bracket considering the ultimate character of nature itself, apart from these interactions, by placing this character beyond conception. But these interpretations, at least the one assumed here, do not claim that this is the ultimate truth of nature. They are only interpretations of the character of our interactions with nature, and the conception of reality as something beyond conception is still a human conception.

On the other hand, short of evolutionary change, our perception and thinking, including concepts, remain classical, in the sense of being refinable, in part mathematically, into those of classical physics. Our mathematics can be abstract, also in the sense of being abstracted from any natural objects as we perceive and conceptualize them in daily experience and, hence, from physical concepts. Mathematics can be used as such even in classical physics, for example, in considering the role of group theory there. This abstractness reflects the fact that our thinking is capable of much more than this type of refinement, defined by the specific needs of classical physics. Our thinking is capable of much more in physics, such as dealing with quantum phenomena by inventing the mathematics necessary for doing so (a type of mathematics also invented in mathematics itself), let alone elsewhere, as in philosophy, literature, or art.

I am not saying the problem or (as one can think of several) problems of the relationships between, or the transition from, QP to CP, or from CP to QP, in fundamental physics is thereby solved. RWR interpretations of quantum phenomena and QT suggest, however, that a different way of thinking concerning these problems (vs. realist ones, more commonly expected or desired) is possible and may be necessary. Moreover, while QM is a fully established theory in low energy regimes, QFT is not entirely complete or without problems, even when dealing (within the standard model) with electroweak and strong forces that are as effective as the electroweak QFT and QCD are. There is, again, no theory for dealing with gravity on quantum scales, and it is not clear if such a theory, if ever found, will be quantum in the present sense.

For the moment, at least in RWR interpretations, one cannot apply the concepts “dead” or “alive” to quantum objects, unless, again, metaphorically, as when the object is destroyed in the experiment, or when we speak of particles that disappear in QFT. Neither a quantum object in any state nor its destruction could be observed as such, in a way one can observe the cat, but only detected by special, quantum, observational instruments. Even the phrase “to be” or concept of “being”, obviously created by human thought, may not be applicable to quantum objects, or the reality ultimately responsible for quantum phenomena, and it does not in RWR interpretations. These considerations also suggest a misleading nature of using the notation $\left.|\psi \right\rangle =\alpha |\left.dead\right\rangle +\beta |\left.alive\right\rangle$, as opposed to something like $\left|\left.\psi \right\rangle =\alpha \right|\left.h\right\rangle +\beta \left.|v\right\rangle$, as related to the probability of finding the cat dead or alive in the cat experiment. The first notation uses, commonly not metaphorically, words designating concepts that cannot apply to quantum objects, as Heisenberg says. In fact, it is difficult to see it as a metaphor, because it represents a possible physical state of an object.

An intriguing recent example of such ontological use of this notation is offered by L. Maccone [52]. It is different from other such cases by arguing that “the superimposed cat has a definite value of a property $\hat{S}$ with eigen state ($|\left.dead\right\rangle \;\pm |\left.alive\right\rangle /\sqrt{2}$, which is … *complementary to* the macroscopic property “being dead or alive”, with the first property interpreted as defining the state of the cat as “neither dead nor alive” or “figuratively” as “both dead and alive” [52] (pp. 7, 1). I cannot consider this argument and, hence, to offer a properly supported assessment of it. I confess, however, that the phrase “neither dead nor alive” gives me a pause. What can it mean beyond our *epistemological* incapacity to attribute either state for such an entity, or that such a statement is inapplicable to a quantum object (as the cat is assumed to be by Maccone before one opens the box)? Maccone, however, does see this phrase, “as defining the state of the cat”, thus giving it an *ontological* physical meaning, which appears to me difficult to substantiate. Instead, speaking of the cat as “neither dead nor alive” appears to me to reflect the difficulty of assuming the cat to be a quantum object in a superposition, rather than, as in the present view, remaining a classical object at any stage of the experiment. In the present view, again, such terms or ordinary language in general does not apply to properly quantum objects. Maccone indeed admits as much: “It is so inconceivable to think of such a “cat” that we do not have appropriate words to describe the situation (except in the language of mathematics) and we resort to somewhat inappropriate ‘dead AND alive’ statements. The reason we do not have experience of such cats is due to the incredible complexity of the experiment necessary to measure the $\hat{S}$ property in a cat” [52] (p. 4). In RWR interpretations the mathematics of QM would not describe the cat as a quantum object either, if the cat were assumed to be, which it is not in the present view. Maccone’s explanation for this situation, *under his assumptions*, is decoherence: “This, in turn, is due to decoherence: the almost unavoidable interaction with the environment of macroscopic systems such as a cat means that some of the cat’s properties (such as $\hat{H}$ [being dead or alive]) are evident, whereas others (such as $\hat{S}$) are not” [52] (p. 4). This is possible. As noted above, however, there is no real experimental evidence that all classical objects contain quantum properties like $\hat{S}$. Nor, contrary to Maccone’s view, is “decoherence” itself “widely accepted” or even “most widely accepted” [52] (p. 4). Moreover, as I argue here, even if one accepted the concept of decoherence, the cat would be outside any decoherence occurring within the process leading to its final state. There is no quantum-to-classical transition in poisoning the cat. It is a purely classical event due to the release of the gas from the flask. All quantum events involved would occur, if they do, before this event, while making it possible.

I might add that the concept of complementarity, as mutual exclusivity of certain properties (such as those specified above as $\hat{S}$ and $\hat{H}$) used by Maccone is different from that of Bohr, as considered earlier in this article. A form of mutual exclusivity is Bohr’s concept. As explained there, however, Bohr’s concept also refers to the possibility of staging, by decision, the experiment considered in two mutually exclusive ways, accompanied by an RWR interpretation. Thus, (A): one can always decide and has a (sufficiently) free choice to do so, to observe and measure, exactly, either the position or the momentum at any given moment in time. On the other hand, (B): by the RWR interpretation assumed by Bohr, either property is only that of a suitable observable part of the instrument used and not (as is in Maccone’s argument) of the quantum object considered, ultimately even at the time of observation. While (B) is part of Bohr’s (RWR) interpretation, (A) is an experimentally established feature, essential to complementarity as Bohr’s concept, and would apply regardless of interpretation. As noted, disregarding (A) leads to a misunderstanding of Bohr’s concept or to defining a different concept, as Maccone’s complementarity is. The cat experiment is staged in only one way, as a single experiment, without a complementary counterpart in Bohr’s sense.

The second notation stated above, $\left|\left.\psi \right\rangle =\alpha \right|\left.h\right\rangle +\beta \left.|v\right\rangle$, which is conventional and unproblematic, and would apply to the present view in the cat experiment, refers to state vectors in a superposition. This superposition allows one to predict definitive classical events or phenomena (which are never in a superposition), such as that of the cat being dead or alive in the cat experiment, but it has no other connections to either (physical) state of the cat. Notations like $\left.|\psi \right\rangle =\alpha |\left.dead\right\rangle +\beta |\left.alive\right\rangle$ may be suggested by Schrödinger’s statement “the ψ-function of the entire system would express this by having in it the living and the dead cat (pardon the expression) mixed or smeared out in equal parts”. They are, however, made even more problematic by not speaking, as Schrödinger still does, of “the ψ-function of *the entire system*”. Seeing the situation in this way allows one to qualify, along the lines considered (Schrödinger himself does not do so) Schrödinger’s statement as follows. One can use this ψ-function for predicting the probability of finding the cat either dead or alive, but without assuming that this ψ-function “would express this by having in it the living and the dead cat (pardon the expression) mixed or smeared out in equal parts”. “The ψ-function of the entire system” would define our expectations concerning the occurrence of two possible outcomes to be observed classically, one of which is a cascade of classically observable events (even if some of them are not actually observed), culminating in the death of the cat.

To make more rigorous the present understanding of the cat experiment and to further support my, hypothetical, surmise that Bohr would have been likely to have a similar understanding of it, I would like to consider part of Bohr’s argument in his reply to EPR. This part concerns the necessity of “discriminating in each experimental arrangement between those parts of the physical system considered which are to be treated as measuring instruments and those which constitute the objects under investigation”, and the question of the so-called “cut” thus arising. According to Bohr:

This necessity of discriminating in each experimental arrangement between those parts of the physical system considered which are to be treated as measuring instruments and those which constitute the objects under investigation may indeed be said to form a *principal distinction between classical and quantum-mechanical description of physical phenomena*. It is true that the place within each measuring procedure where this discrimination is made is in both cases largely a matter of convenience. While, however, in classical physics the distinction between object and measuring agencies does not entail any difference in the character of the description of the phenomena concerned, its fundamental importance in quantum theory … has its root *in the indispensable use of classical concepts in the interpretation of all proper measurements*, even though the classical theories do not suffice in accounting for the new types of regularities with which we are concerned in atomic physics. In accordance with this situation there can be no question of any unambiguous interpretation of the symbols of quantum mechanics other than that embodied in the well-known rules which allow us to predict the results to be obtained by a given experimental arrangement described in a totally classical way. [12] (p. 701; second emphasis added)

It is important to avoid two common misunderstandings of this and related statements by Bohr. The first concerns observational instruments. This misunderstanding tends to disregard that, as discussed in Section 2 and Section 3, Bohr only refers to the necessity of the classical description of the *observable* parts of measuring instruments, while assuming that they also have quantum parts through which they interact with quantum objects. The second misunderstanding concerns quantum objects. Bohr’s statement does not mean that, while observable parts of measuring instruments are described by CP, the independent behavior of quantum objects is *represented* by the formalism of QM. This view would conflict with the RWR interpretation held by Bohr in his reply to EPR. It has been adopted by some, sometimes under the heading of “the Copenhagen interpretation”, beginning with Dirac’s and, especially, von Neumann’s influential classic studies, published in 1930 and 1932, respectively [19,53]. Both books, moreover, assume a classically causal independent behavior of quantum objects, with probability brought in only by measurement. (See [54] for an instructive critique of von Neumann’s interpretation). This was, however, not Bohr’s view, especially at this stage of his thinking in 1935, or even almost immediately after the Como lecture of 1927. (The latter may be seen as having adopted, ambivalently, this type of view and having influenced both Dirac and von Neumann in this regard [28] (pp. 198–211)). In the passage just cited, Bohr only says that CTs cannot account for how quantum phenomena (physically described classically) come about or predict the data observed. He does not say that the independent behavior of quantum objects or objects “under investigation” (which may not be strictly quantum but must contain properly quantum objects) is represented by the formalism of QM. As explained earlier, in Bohr’s view, the “symbols” of QM only have a probabilistically predictive role, without, by the Heisenberg postulate, offering a representation of how quantum phenomena come about, while quantum phenomena are represented by CP, by the Bohr postulate. Thus, while predicting the data observed in quantum phenomena, the formalism of QM is otherwise dissociated from both the ultimate nature of reality responsible for quantum phenomena and quantum phenomena themselves.

The circumstance that “the place within each measuring procedure where this discrimination is made is … largely a matter of convenience” is related to the arbitrariness of the cut, often called the Heisenberg cut or the Heisenberg–von Neumann cut, because Heisenberg and von Neumann favored the term, not used by Bohr. Bohr qualifies this claim, and this qualification is important, including in the context of the cat experiment. While “it is true that the place within each measuring procedure where this discrimination is made is … largely a matter of convenience”, it is true only largely but not completely. This is because “in each experimental arrangement and measuring procedure we have only a free choice of this place within a region where the quantum-mechanical description of the process concerned is effectively equivalent with the classical description” [12] (p. 701). Accordingly, it is how we set up a quantum experiment that defines what is “the object under investigation” in this experiment, while the quantum nature of this experiment is defined by the ultimate, RWR-type reality, whose existence is independent of any experiment and is manifested or “symbolized” by Planck’s *h* [20] (v. 1, p. 53).

As, in RWR interpretations, part of the ultimate reality responsible for quantum phenomena and, as such beyond representation or even conception, quantum objects and quantum parts of the instruments interacting with quantum objects, are never on the observation side of the cut. Hence, they can never serve as observational instruments either. All observable properties are those of the observable parts of the instruments and, along with these parts, are described classically, by the Bohr postulate, while appearing, as such properties, under the impact of quantum objects. As associated with the discrimination between what is considered the object under investigation and what is the observational instrument, the cut and its (within certain limits) shifting nature need not imply that CT is a special, limited form of QT. In the present view, CT and QT are different theories that deal with two different types of objects, even though classical objects are composed of quantum objects or the same ultimate reality.

The cut reflects the possibility of placing some classical parts of the overall arrangement in a quantum experiment (including the cat experiment) on either side of the cut, as “an object under investigation”, concerning which predictions can be made by QM. As I argue, however, for the experiment to have quantum effects, the arrangement must include a properly quantum object (like a particle in radioactive decay and the decaying atom in the cat experiment), unobservable, regardless of interpretation, and beyond conception, invisible to thought, in (strong) RWR interpretations. The cat is, on the other hand, always visible at least to our mind’s eye, even when, while inside the box, not actually available to our sense perception. A quantum object is never available to such a perception, and one always needs an experimental device capable of interacting with this object to have a quantum effect. This effect is manifested in a classically observed phenomenon or event, such as the cat being alive or dead in this case, rather than being a quantum effect. It is preceded and made possible by another classically observable (even if not actually observed) actual event, the breaking of the flask, due to the discharge of the counter tube. This discharge is a more proper quantum effect, due to the interaction between the tube and the emitted particle as a quantum object. This interaction is quantum, while the rest is the chain of classical events triggered by it. As noted, one need not use CT formally (or quantitatively) to represent these classical processes and events, but one, in principle, could. On the other hand, predicting the emission of the particle that leads to this classical chain requires QT. It is because the object considered can contain both a classical and a quantum object, on the object side of the cut, Bohr, in the passage cited above, calls it the “object under investigation”, rather than the quantum object. While they can also be objects under investigation, properly quantum objects are objects that fully belong to the ultimate reality responsible for quantum phenomena. As such, they are always on the object, and never observation, side of the event. Hence, as stated, a quantum object can never be an instrument. Other parts of the objects under investigation in quantum experiments may be classical, such as the cat or everything inside the box, except the quantum part of the counter tube that can interact with the emitted particle. While it can be *part* of an object of investigation in a quantum experiment and treated by quantum means, if considered by itself, a classical object cannot be so treated. It can only be treated by CP.

On the other hand, in certain circumstances, a quantum object considered by itself could be *treated* as a classical object, but *without ever being* a classical object. Thus, as when an electron is far enough from the nucleus (for large quantum numbers), its behavior can be treated classically. This is, however, an idealized approximation that disregards the possible quantum effects of this behavior. As Bohr noted, connecting this situation to “mechanical pictures” and “classical pictures” and, thus, to visualization:

[I]n the limit of larger quantum numbers where the relative difference between adjacent stational states vanishes asymptotically, mechanical pictures of electronic motion may be rationally utilized [by the correspondence principle]. It must be emphasized, however, that this connection cannot be regarded as a gradual transition toward classical theory in the sense that the quantum postulate [representing the essential discontinuity of quantum phenomena] would lose its significance for high quantum numbers. On the contrary, the conclusions obtained from the correspondence principle with the aid of classical pictures depends just upon the assumptions of the conception of stationary and of [discrete] individual transition processes are maintained even in this limit. [20] (v. 1, p. 85)

This statement confirms Bohr’s view of classical objects and processes as available, visible, to thought or even our immediate phenomenal perception, and of quantum objects and their behavior as no longer available to our general phenomenal intuition, if at this point (in 1927) not yet invisible to thought. Bohr, again, only came to understand the situation in these, strong RWR terms, by the late 1930s. The behavior in the limits of large quantum numbers can be treated for practical purposes as those of classical objects. This treatment is, however, merely a workable approximation of the ultimate nature of the reality responsible for what is, thus, observed, and one requires a measuring instrument for this observation. At the bottom, one still deals with the combination of stationary states and discontinuous quantum jumps. These states are too close to each other for this combination to be detected, but one would register these states (as invisible to thought, electrons or the photons they emit still cannot be “seen”) by “zooming” on them if one had an instrument to do so. Any such instrument would, however, need to be able, by interacting with electrons or “emitted” photons, to register properly quantum effects. Technically, an “emission”, too, is a classical concept, which cannot represent how photons are “emitted” because nothing can in RWR interpretations. All we can see are traces of photons, or what we assume to be photons, traces manifested, visible, in spectra. Similarly, as a quantum object, a macroscopic quantum object (still defined as such by its microscopic quantum constitution), such as a Josephson device, can only be detected as quantum by means of a suitable instrument. Otherwise, as noted, it will be observed as a classical object and as such, as something (two superconductors standing in a lab) available to our immediate perception. In sum, a quantum object, if considered by itself, can be treated by both, always QT and sometimes CT, without being a classical object; by contrast, a classical object, if considered by itself, can only be treated by CT, unless it is linked to a properly quantum object, in which case the combined object can be treated by QT.

In QP, on the one hand, there is always a discrimination between an object and an instrument, and, on the other, their indivisibility or wholeness of quantum phenomena, as, in Bohr’s terms, its closed nature, from which one can never extract the object itself at the time of measurement. Any investigation in QP involves this combination, which is that of what is visible to thought, via observational instruments, and can be communicated unambiguously, and what is invisible to thought and cannot be communicated unambiguously. This situation sharply contrasts with that of CP or relativity, where the role of observational instruments can be neglected or controlled and where, as a result, one always deals with what is visible to thought and unambiguously communicable. If the object under investigation is classical, like the cat in the cat experiment, it can always be considered independently apart from quantum experiments and discussed unambiguously. There is never any ambiguity in assessing the cat as an independent object in the cat experiment but only two unambiguously defined possibilities, each visible to our mind’s eye, of the cat being either dead or alive. The probability of either is defined by the ψ-function, which, however, is associated essentially with the atomic decay and only secondarily with the state, always classical, of the cat. A cat, inside or outside the box, is always a cat, dead or alive. As such it can only be seen as a physically classical part of the arrangement, before the interaction with the particle emitted by an atom, which particle can never be observed as such. The cat can be on both sides of the cut (on the side of the object when connected to a properly quantum object), but the radioactive decay or the particle emitted by it can only be on the side of the object, and never the observation side. This emission occurs regardless of the cat in the box, or the box, the flask, and other parts of the concoction used, all of which are classical objects. The cat could be removed from the box without affecting the possible quantum event of the particle emission. The counter tube is the only classical object that interacts with the particle, which interactions enable one to register this emission (if it occurs).

Indeed, one need not see the opening of the box as the proper quantum experiment in the arrangement. Instead, this experiment is the shattering of the flask, which occurs (if it does) before the box is opened. Then the outcome of this experiment leads to the event that classically affects the cat, killing it or not, if it does not occur. The cat is more like an “agent” than part of the observational instrument used. In this regard, the cat is more akin to Wigner’s friend in a related thought experiment proposed by E. Wigner in part to sharpen some of the aspects and paradoxes (or what is so seen) of QM vis-à-vis the cat experiment [55]. Wigner’s experiment can be considered along the lines of the present argument concerning the cat experiment. This would, similarly, allow one to avoid some of the paradoxes arising if one considers “the friend” as a quantum object rather than as a classical object combined with the arrangement of the experiment the friend performs, an arrangement that involves properly quantum objects. The case would, however, require a separate analysis, given the additional complexities it contains, especially the role of the consciousness of a human observer. I should only reiterate that, as in the cat experiment, the whole arrangement, by including a properly quantum object, along with “the friend”, can and even must be treated as a quantum system, concerning “Wigner’s” prediction. An instructive analysis of the experiment in a modified version (and a critique of recent approaches to the experiment along similar lines [56,57]) from the quantum Bayesian (QBist) perspective is offered in [58]. This analysis, which deals with “Wigner’s” quantum predictions, could be seen consistently with the present view, different from QBism, although sharing with it a de Finettian view of probability. My main point now is that the present view implies the necessity of having a properly quantum object to use QT in predictions concerning systems involving classical objects, such as the cat or “the friend”. Otherwise, only CT can be used for such predictions. By contrast, in dealing with properly quantum objects, both, *always*, QT and, *sometimes*, CT can be used. Either way, in RWR interpretations, quantum objects or, if one adopts the Dirac postulate, which only allows one to speak of quantum objects (as RWR entities) at the time of observations, the reality ultimately responsible for quantum phenomena remains invisible to thought.

## 5. Conclusions

Bohr saw the existence, at least a possible existence, of a reality invisible to thought, as ultimately responsible for quantum phenomena, as “an epistemological lesson of quantum mechanics” [20] (v. 3, p. 12). At least, this was an epistemological lesson of his interpretation, to which the present interpretation adds the Dirac postulate. Arguably, however, physics cannot teach us its epistemological lessons otherwise than by an interpretation. There is merely more consensus concerning the interpretation of CP and relativity as realist theories, although this consensus is not entirely unanimous. It has been questioned whether the mathematical architecture of relativity corresponds to the architecture of nature, as opposed to serving as a model for correct predictions concerning relativistic phenomena [59]. In this case, these predictions are deterministic, as opposed to the probabilistic predictions of QT, even in dealing with most elementary individual quantum phenomena. This is a fundamental difference due to the impossibility, in principle, of controlling the interference of observational instruments with the quantum object under investigation, regardless of interpretation. When it comes to QM and QFT, the proliferation of diverse (even incompatible) interpretations has been relentless and continues, along with debates concerning them, with an undiminished intensity and no end in sight.

But then, the stakes are high: our understanding of matter and thought alike. Defining the ultimate reality considered in QP beyond the reach of thought, RWR interpretations, beginning with Bohr’s ultimate interpretation, are the most radical manifestations of “*der Kopenhagener Geist der Quantentheorie* [the Copenhagen spirit of quantum theory]”, as Heisenberg called it [44] (p. iv). This spirit is, again, not the same as “the Copenhagen interpretation”, which term may designate interpretations that are difficult to see as being in this spirit, as it was understood by Heisenberg or Bohr.

I return, in closing, to Denmark, first, not to that of Bohr and Copenhagen but that of Shakespeare’s *Hamlet* and Elsinore three centuries earlier, and the lines of the play used as my epigraph:

Hamlet [commenting on his dead father]:

My father—methinks I see my father.

Horatio:

Where, my lord?

Hamlet:

In my mind’s eye, Horatio.

(*The Tragedy of Hamlet*, *Prince of Denmark* [60] (Act 1, Scene 2, ll. 183–185))

The reason for Horatio’s puzzlement is that he saw the ghost of Hamlet’s father and wondered if perhaps Hamlet had already seen the ghost, which Hamlet’s response proves not to be the case. Hamlet’s encounter with the ghost of his father is yet to come. At stake in this scene is Hamlet’s image of his father in his mind’s eye. The image shadows Hamlet and the play from beginning to end. I am not concerned here with much discussed psychological, such as psychoanalytic, implications of this image. My point involves the capacity of our thoughts to create images of the world and its objects. Shakespeare explores this capacity in *Hamlet* and other works. No less remarkable, however, is our thought’s capacity to think that which is beyond thought, is invisible to thought and, hence, has no image in our mind’s eye, at a given moment in time, possible to be overcome, or forever beyond the reach of thought. Anticipating Kant’s or even Bohr’s more radical (RWR) thinking, Shakespeare realized this capacity, as suggested by Hamlet’s comment to Horatio after his encounter with the ghost:

Horatio: O day and night, but this is wondrous strange!

Hamlet: And therefore as a stranger give it welcome.

There are more things in heaven and earth, Horatio,

Than are dreamt of in your philosophy.

(*The Tragedy of Hamlet, Prince of Denmark* [60] (Act I, Scene 4, ll. 165–166))

Some editions have “our philosophy”. “Your philosophy” makes Hamlet more suspicious of philosophy and its “dreams”, rather than a sufficient capacity to deal with the reality of nature or mind. These lines also tell us that there are things in nature and our minds that we cannot dream of or otherwise see in our mind’s eye, consciously or unconsciously. There is also Shakespeare’s play on a “stranger”, with the comparative form of “strange” referring to “strangeness”, and the noun “stranger” referring to an unknown person, perhaps an unknown visitor to one’s house.

In Denmark, three centuries later, Bohr is reported to have replied, after the rise of QP but before QM, to H. Høffding’s question “Where can the photon be said to be?” with “To be, to be, what does it mean to be?” (cited in [61] (p. 131)). Bohr might have been echoing the most famous sentence of Shakespeare’s *Hamlet*, “To be, or not to be, that is the question” (Act 3, ll. 1749), realizing that in QP one might have to ask first “What does it mean to be?” (Hamlet’s monologue is, too, about much more than deciding to live or die, and may even be read as asking Bohr’s question). Høffding’s and Bohr’s questions are still unanswered and, in Bohr’s ultimate view, are unanswerable, when it comes to quantum objects, such as photons. Such questions as “Where can something be said to be?” or “When had something happened?” can only be asked about quantum phenomena, observed in our instruments, and as such visible to our mind’s eye or even to our immediate perception. Nature has no photons or electrons, any more than being or reality, including that of the RWR-type. One might think, and physicists often do, that one sees a “photon” in the mind’s eye. In strong RWR interpretations, however, whatever one sees in one’s eye cannot be a *photon,* no matter how it is imagined. It cannot be anything that belongs to the ultimate reality responsible for quantum phenomena, such as a spot on a photographic plate, that we *see* and associate with the effect of what we call a photon that collided with the plate. Still, as stated from the outset, even a reality beyond thought is only an idea created by thought. Of course, our capacity for thought is created by our brains, even if we do not know how, because, as things stand now, we do not know how our brains neurologically create thinking or what thinking is physically. Our thoughts are still created by matter and nature. This is not the same as saying, as some do, that nature uses these thoughts to think about itself. Nature allows *us* to create thoughts and to use them when considering our interactions with nature by means of technology, beginning with that of our bodies, and our thoughts. These interactions also enable us to conceive of and know part of nature, even in its ultimate constitution, possibly as something that is beyond the reach of thoughts and is invisible to thoughts. What these interactions—possibly with something in nature that we cannot see even in our mind’s eye—enable us to *see* in quantum phenomena is “wondrous strange”, akin to what we see in quantum experiments, famous for their strangeness. Perhaps, however, we should welcome this strangeness, even if it is made possible by something invisible to thought, as a guest in the house of physics, rather than worry about it, as many still do, as an unwelcome ghost that has taken residence there and will eventually leave sooner or later. We should not be too surprised either if stranger ‘strangers’ visit the house of physics in the future.

## Data Availability

Data is contained within the article.
